# Establishment of macaque trophoblast stem cell lines derived from cynomolgus monkey blastocysts

**DOI:** 10.1038/s41598-020-63602-7

**Published:** 2020-04-22

**Authors:** Shoma Matsumoto, Christopher J. Porter, Naomi Ogasawara, Chizuru Iwatani, Hideaki Tsuchiya, Yasunari Seita, Yu-Wei Chang, Ikuhiro Okamoto, Mitinori Saitou, Masatsugu Ema, Theodore J. Perkins, William L. Stanford, Satoshi Tanaka

**Affiliations:** 1 Department of Animal Resource Sciences, Graduate School of Agricultural and Life Sciences, The University of Tokyo, Toky, 113-8657 Japan; 2 The Ottawa Hospital Research Institute, Ottawa, ON K1H 8L6 Canada; 3 Department of Stem Cells and Human Disease Models, Research Center for Animal Life Sciences, Shiga University of Medical Sciences, Shiga, 520-2192 Japan; 4 Department of Veterinary Physiology, Graduate School of Agricultural and Life Sciences, The University of Tokyo, Tokyo, 113-8657 Japan; 5 Department of Anatomy and Cell Biology, Graduate School of Medicine, Kyoto University, Kyoto, 606-8501 Japan; 6 Japan Science and Technology (JST), Exploratory Research for Advanced Technology (ERATO), Kyoto, Japan; 7 Institute for Advanced Study of Human Biology (WPI-ASHBi), Kyoto University, Kyoto, 606-8501 Japan; 8 Center for iPS Cell Research and Application (CiRA), Kyoto, 606-8507 Japan; 9 Department of Cellular and Molecular Medicine, University of Ottawa, Ottawa, K1H 8M5 Canada

**Keywords:** Stem cells, Reproductive biology

## Abstract

The placenta forms a maternal-fetal junction that supports many physiological functions such as the supply of nutrition and exchange of gases and wastes. Establishing an *in vitro* culture model of human and non-human primate trophoblast stem/progenitor cells is important for investigating the process of early placental development and trophoblast differentiation. In this study, we have established five trophoblast stem cell (TSC) lines from cynomolgus monkey blastocysts, named macTSC #1-5. Fibroblast growth factor 4 (FGF4) enhanced proliferation of macTSCs, while other exogenous factors were not required to maintain their undifferentiated state. macTSCs showed a trophoblastic gene expression profile and trophoblast-like DNA methylation status and also exhibited differentiation capacity towards invasive trophoblast cells and multinucleated syncytia. In a xenogeneic chimera assay, these stem cells contributed to trophectoderm (TE) development in the chimeric blastocysts. macTSC are the first primate trophoblast cell lines whose proliferation is promoted by FGF4. These cell lines provide a valuable *in vitro* culture model to analyze the similarities and differences in placental development between human and non-human primates.

## Introduction

The placenta is an organ that forms a maternal-fetal junction and carries out various physiological functions such as facilitating the supply of nutrition to the fetus, exchange of gases, and removal of wastes from the fetus. In human and non-human primates, this multifunctional organ is characterized by the presence of three major placenta-specific cell-types, including proliferative cytotrophoblasts (CTBs), multinucleated syncytiotrophoblasts (STBs), and invasive extravillous trophoblasts (EVTs). CTBs include two different subtypes, villous CTBs and cell column trophoblasts. They are recognized as progenitor cell populations because villous CTBs and cell column trophoblasts differentiate into STBs and EVTs, respectively, during placentation^1,2^. When isolated and cultured *in vitro*, human CTBs cannot maintain an undifferentiated state and eventually differentiate into STBs^3^. Therefore, the establishment of an *in vitro* culture model for human and non-human primate trophoblast stem/progenitor cells is essential for investigating the process of early placental development and trophoblast differentiation in the primate.

The HTR8/SVneo cell line, developed from primary EVTs, is a widely used model for human trophoblast *in vitro;* although, HTR8/SVneo cells express OCT4 and NANOG, the embryonic stem cell (ESC) markers^4,5^. In human blastocyst, OCT4 is detected in some cells of trophectoderm (TE), while the expression of NANOG is strictly restricted to the inner cell mass (ICM)^6^, hence questioning the suitability of HTR8/SVneo cells. The BeWo cell line derived from human choriocarcinoma has also been used as a human trophoblast *in vitro* model^7^. The BeWo has syncytialization and invasion abilities^8,9^; however, choriocarcinoma cells might represent unnatural features compared to endogenous trophoblasts. Trans-differentiation of human ESC to trophoblast-like cells by BMP4 treatment has also been adopted^2^. However, the adequacy of trans-differentiation system remains somewhat controversial, since the gene expression profile of the resulting trophoblast-like cells do not resemble that of primary trophoblast cells, moreover they express other cell lineage markers^2,10^. Recently, the trans-differentiation protocol from human ESC to trophoblast-like cells was improved in so-called BAP treatment^11^. This newer treatment succeeded to suppress the upregulation of mesoderm markers including T (Brachyury)^11^, although the difference in global gene expression profile between trans-differentiated human ESC and primary trophoblast remains^12^.

In mice, the trophoblast stem cells, which have the potential to differentiate both *in vivo* and *in vitro* into all trophoblast subtypes, were established from the preimplantation blastocysts and extraembryonic ectoderm of post-implantation embryos in the presence of fibroblast growth factor 4 (FGF4)^13^. This useful *in vitro* model has revealed the underlying mechanisms of trophoblast differentiation and placental development. Attempts to establish human TSCs by employing the same strategy used for mouse TSCs has been unsuccessful^14^, suggesting that establishment of *in vitro* human TSCs may depend on certain exogenous factors, which remains different from mouse TSCs. Okae *et al*., recently succeeded in establishing human TSC lines from blastocysts and early gestation placentae, highlighting the essential roles of epidermal growth factor (EGF) and a WNT activator in the establishment and maintenance of human TSCs^15^. This study clearly showed that an exogenous FGF4 is not required for the proliferation of human TSCs, though the direct effect of FGF4 on human TSCs was not examined.

Although both human and mouse have the discoidal hemochorial placenta, there are substantial differences between these two species in placental formation processes after implantation^16,17^. Successful establishment of human TSCs employing a group of exogenous factors, completely different from mouse TSCs, raised questions on whether this dependency on different factors reflect the species-specific differences between mice and humans, or if it could be generalized as cellular differences between rodents and primates^15^. Establishment of TSCs from non-human primates will offer valuable insights to address this important question. Previous reports show the establishment of TSC lines from rhesus monkey blastocysts^18^. Although these cell lines were capable of differentiating into both invasive cells and multinucleated cells; they also expressed the ICM/ESC marker, OCT4, and the marker for STBs, chorionic gonadotropin (CG)^18,19^, without induction of differentiation, making it uncertain if these cells are genuine TSCs. Accordingly, the establishment of trophoblastic stem/progenitor cell lines from a non-human primate, whose placental structure is similar to that of humans, remains an important issue. To address this, we aimed to derive cell lines from the cynomolgus monkey blastocysts, and successfully obtained stable proliferating trophoblastic cell lines exhibiting differentiation potential, by adopting the modified culture conditions for mTSCs without using EGF and WNT activator.

## Results

### Establishment of cynomolgus monkey trophoblastic cell lines from blastocysts

**Figure 1:**
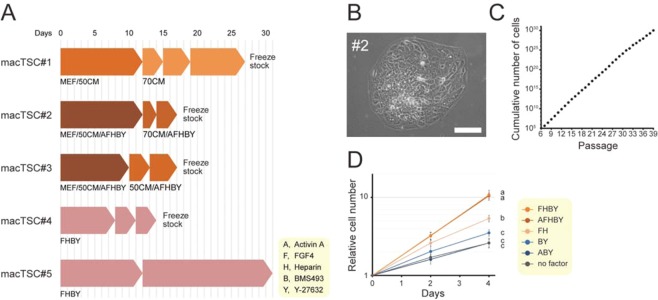
Establishment of cynomolgus monkey trophoblast stem cell lines (macTSC) from blastocysts. (**A**) Schematic representation of the cell culture conditions and experimental procedure. Blastocysts #1-3 were initially co-cultured with mouse embryonic fibroblasts (MEF) that was substituted by 50 or 70% MEF-conditioned medium (50CM, 70CM) as indicated after the first passage. Abbreviations of the exogenously added reagents are shown. The other two blastocysts (#4 and #5) were cultured without MEF in the TS medium supplemented with FHBY. The fat arrow-like shapes depict each passage. (**B**) The appearance of the obtained cell line macTSC#2. Scale bar = 100 µm. (**C**) Growth of macTSC#2 cells over 132 days (from passage number 6 to 39) of culture in the AFHBY condition. At each passage, cells were harvested, counted, and seeded at 1 × 10^5^ per 35-mm dish in triplicate. A mean value of triplicate was used to calculate the theoretical cumulative number. (**D**) Growth of macTSC#2 cells in different culture conditions. The number of cells normalized to that at day 0, and arbitrarily set as 1. The mean values (±SD) of triplicates of biological duplicates are shown. Statistical analysis was done by the Tukey-Kramer test.

We attempted to establish trophoblastic cell lines from cynomolgus monkey blastocysts by adopting the conditions used for the establishment and maintenance of mouse TSC, with slight modifications. In the original protocol, mouse TSC lines are established in the presence of FGF4, heparin, and mouse embryonic fibroblast cells (MEF)^13,20^. For the maintenance of mouse TSCs, MEF can be replaced by the MEF-conditioned medium (MEF-CM) or by activin A^20,21^. We, therefore, co-cultured a single cynomolgus monkey blastocyst (#2) and MEF in the TS medium supplemented with 50% MEF-CM, activin A, FGF4, and heparin (abbreviated as 50CM/AFH in Fig. 1A). In addition to AFH; BMS493, a pan-RAR inverse agonist; and Y-27632, a ROCK inhibitor (abbreviated as BY in Fig. 1A) were also added because these reagents appeared to facilitate maintenance of mouse TSCs in a serum-free, knockout serum replacement (KSR)-supplemented condition (manuscript in preparation). Another blastocyst (#1) was also co-cultured with MEF, but without AFHBY as a control. Both blastocysts attached to the surface of tissue culture dish displaying cellular outgrowth by the 5^th^ day of culture, although the embryos did not hatch completely (Fig. S1A). The blastocyst outgrowths were passaged to expand on the 12^th^ day of culture, and 70% MEF-CM was added (70CM) instead of co-culturing with MEF. As a result, a stable growing cell line forming a flat monolayer of colonies with epithelial cell-like appearance, named macTSC#2, was obtained from blastocyst #2 after another passage in AFHBY-containing media (Fig. 1A,B). The macTSC#2 cells steadily grew for at least 132 days (4 days × 33 passages) without an apparent reduction in their growth potential (Fig. 1C). A cell line, indistinguishable from macTSC#2, albeit with slower cell growth, was obtained unexpectedly from the blastocyst without AFHBY and named macTSC#1 (Figs. 1A and S1B).

To elucidate the factors required for the growth of macTSC#2, we cultured the cells with several combinations of factors used in the AFHBY condition, but in the absence of MEF. The combination of FGF4 and its co-factor heparin (FH) promoted cell growth, whereas activin A was dispensable. Removal of FH or all the factors slowed the growth rate of cells, but cells grew stably unlike mouse TSCs (Figs. 1D and S1C). We also observed a positive effect of BMS493 and Y-27632 (BY) on cell growth and decided to use the FHBY condition for routine maintenance of macTSC lines. Under this condition, growth of macTSC#1 was also stimulated (Fig. S1E), and 49.0% and 40.7% of macTSC#1 and #2 cells, respectively, remained euploid after 21 – 30 (#1) and 20 – 35 (#2) passages (Fig. S1D,F).

The adequacy of the FHBY condition was verified by culturing another set of blastocysts (#3-#5, Fig. 1A). While the establishment of macTSC#3 cell line was done using the same initial condition as was used for macTSC#2, thereby demonstrating the reproducibility of the procedure, the two other macTSC lines; namely, macTSC#4 and #5, were obtained using the FHBY condition, showing the dispensability of MEF. Cells of macTSC#3-#5 lines also had an indistinguishable appearance from macTSC#1 and #2 (Fig. S1B).

### Trophoblast-like DNA methylation profile of macTSCs

**Figure 2:**
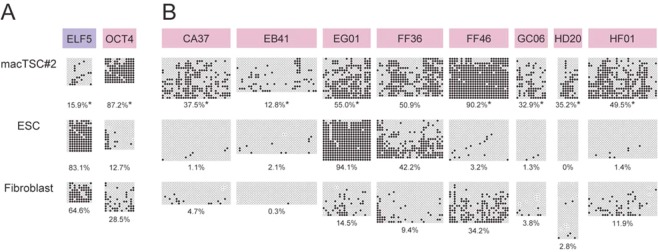
Characterization of macTSC#2 by DNA methylation profile. *(A* and *B)* DNA methylation status of *ELF5* and *OCT4* promoter regions (**A**) and the T-E T-DMRs (**B**; CA37, EB41, EG01, FF36, FF46, GC06, HD20, HF01, and OCT4) in macTSC#2, ES cell (ESC) and embryonic fibroblasts of cynomolgus monkey were analyzed by bisulfite sequencing. Open and filled circles represent unmethylated and methylated cytosines, respectively. Overall methylation percentage (the number of methylated CpGs per number of total CpGs) is shown under each part. *p < 0.01 between macTSC#2 and ESC (nonparametric two-tailed Mann-Whitney U-test).

Recently, Lee *et al*. proposed a criteria that involves hypomethylation of the *ELF5* promoter to define human early trophoblast cells^22^. We also identified differentially methylated genomic regions, with higher methylation in the trophoblast cell lineage than in the embryonic cell lineage in mice and humans, and named such regions trophoblast-embryonic tissue-dependent and differentially methylated regions (T-E T-DMRs)^23^. To characterize macTSCs, we analyzed the DNA methylation status of the *ELF5* promoter, and the T-E T-DMRs by bisulfite sequencing (Fig. 2). The *OCT4* promoter region was hypermethylated, while the *ELF5* promoter was hypomethylated in macTSC#2 (Fig. 2A), demonstrating that macTSC possess trophoblastic DNA methylation status. Seven out of nine T-E T-DMRs (i.e., CA37, EB41, FF46, GC06, HD20, HF01, and OCT4) showed significantly higher methylation status in macTSC#2 compared to both ESC^24^ and embryonic fibroblast cells of cynomolgus monkey (Fig. 2B). The FF36 region was methylated moderately in macTSC#2; however, this region was methylated similarly in ESCs, unlike in mouse and human ESC. The EG01 region was hypermethylated in ESC, again unlike mouse and human ESC. Analysis of macTSC#1 gave similar results (Fig. S2A,B). These epigenetic features in T-E T-DMRs also supported that macTSCs were of trophoblastic lineage. Thus, the bisulfite sequencing analysis revealed a DNA methylation profile of macTSCs consistent with their trophoblastic origin.

### Expression of miRNAs of primate-specific chromosome 19 miRNA cluster (C19MC)

**Figure 3:**
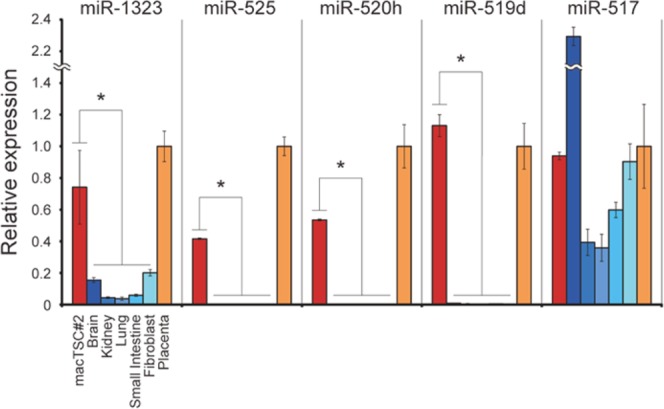
The relative expression levels of five C19MC miRNAs in macTSC#2, brain, kidney, lung, small intestine, embryonic fibroblasts, and placenta of cynomolgus monkey. The mean expression level (±SD) was normalized to that of U6 snRNA. Values of each miRNA in placenta was arbitrary set as 1. *p < 0.05 (technical triplicate). The Tukey-Kramer test was used for statistical analysis.

Another criterion by Lee *et al*. for defining early human trophoblast was the high expression of C19MC miRNAs^22^. These miRNAs are expressed almost exclusively in the human placenta, but also in ESC and some other tissues^25–27^. C19MC miRNAs are highly conserved in the macaque genome, and some of them were expressed in first trimester and term placenta^28^. We detected the expression of all C19MC miRNAs examined in macTSC#2. Four of them (miR-1323, miR-525, miR-520h, and miR-519d) showed significantly higher expression in macTSC#2 than in somatic tissues (Fig. 3), in which these three miRNAs were almost undetectable. Even though the expression of miR-517s (the primer set detected all subtypes of miR-517) was detected in somatic tissues, it was also detected in macTSC#2 at a comparable level to that in the placenta. Expression pattern of those C19MC miRNAs was detected at a similar level in macTSC#2 as compared to the other macTSC lines (Fig. S3). These results suggested that macTSCs, derived from cynomolgus monkey blastocysts, express members of the primate trophoblast specific C19MC miRNAs.

### Expression of genes related to placental development

**Figure 4:**
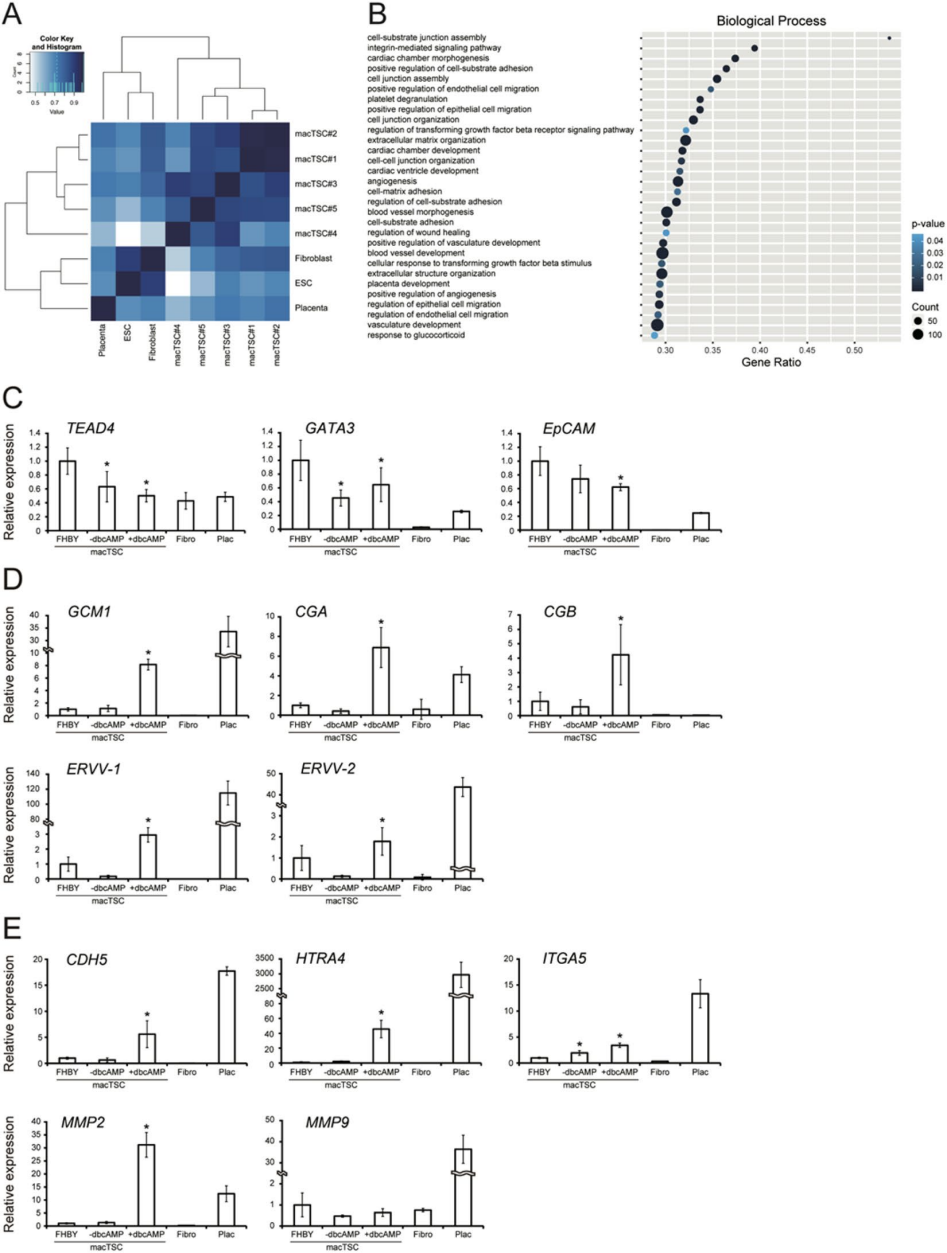
Gene expression profiling of macTSC lines. (**A**) Heatmap was drawn by the values of correlation coefficients using the RNA-seq datasets of macTSCs, ESC, fibroblast, and cynomolgus monkey placenta. (**B**) Enriched GO terms associated with the genes with >2-fold expression in all macTSCs compare to that in ESC. PANTHER (www.pantherdb.org) was used for GO analyses. Gene ratio, the ratio of the analyzed genes in the gene set of the GO term; Count, the number of analyzed genes in the GO term. *P*-values were calculated using Fisher's exact test with Bonferroni correction. (**C**,**D**,**E**) qRT-PCR for analyzing the expression of CTB (**C**), STB (**D**) and EVT (**E**) markers. The mean values (±SD) of technical replicates for biological duplicates, normalized by the expression of *ACTB*, were shown relative to those of cells maintained under the FHBY condition (arbitrarily set as 1). −dbcAMP, basal TS medium without FHBY and dbcAMP; +dbcAMP, basal TS medium with 1 mM dbcAMP. *p < 0.05 vs. FHBY (Two-tailed Student's *t*-test).

To explore similarities among macTSC lines, and their transcriptomic characteristics, we performed RNA-seq analyses. First, we compared the correlation of gene expression profiles of all macTSC lines with ESC, fibroblast, and placenta. As shown in Fig. 4A, all macTSCs formed a close cluster apart from the ESC, fibroblast, and placenta, suggesting that all macTSC lines have similar gene expression profiles even though they were established under different culture conditions. Gene ontology (GO) term enrichment analysis of the genes with >2-fold higher expression in all macTSC lines than in ESC revealed the terms associated with angiogenesis, positive regulation of epithelial cell migration, and placenta development (Fig. 4B). Importantly, some genes such as *KRT7*, *GATA3*, *TFAP2C*, *VGLL1*, and *HAND1*, which are known to be highly expressed in trophoblasts, were also highly expressed in macTSCs compared to ESCs (Table S1). In contrast, genes with >2-fold lower expression in macTSC lines than in ESC were associated with somatic cell differentiation (Fig. S4). These results collectively indicated that macTSCs are cells of trophoblast origin.

Recent reports showed human *VGLL1* gene expression in proliferative, undifferentiated CTBs residing in the early human placenta^29^. The robust expression of *VGLL1* in macTSCs thus implied that the macTSCs might have the potential to differentiate. To test this, we treated the cells with dbcAMP in the absence of FHBY, and analyzed gene expression of each trophoblast subtype markers, since an increase in cellular cAMP is known to induce differentiation of human trophoblast cells^8,30^. First, we analyzed the expression of CTB markers by qRT-PCR (Fig. 4C). The expression levels of *TEAD4*, which is widely used as an undifferentiated trophoblast marker^31^, were downregulated by the FHBY removal regardless of the presence of dbcAMP. *GATA3*, known as a trophoblast marker^22^, was also downregulated. On the other hand, *EpCAM* expression was not reduced significantly by the removal of FHBY, but significantly downregulated by the dbcAMP treatment. In contrast, STB markers, *GCM1*, *CGA*, and *CGB* were upregulated significantly by dbcAMP treatment (Fig. 4D). In addition, expression of the putative fusogenic endogenous retroviral genes, *ERVV-1*, and *ERVV-2*^32^, was also increased (Fig. 4D). As shown in Fig. 4E, *HTRA4*, *ITGA5*, and *CDH5*, which are used as EVT markers, were also upregulated significantly by dbcAMP treatment. One of the invasiveness-related matrix metalloproteinase genes, *MMP2*, was also induced by dbcAMP treatment. Although RNA-seq indicated upregulation of another member of *MMP* gene family, *MMP9*, in all the five macTSCs (Table S2), the expression of *MMP9* did not change by culture conditions based on the primer set used.

### Differentiation of multinucleated STBs from macTSC

**Figure 5:**
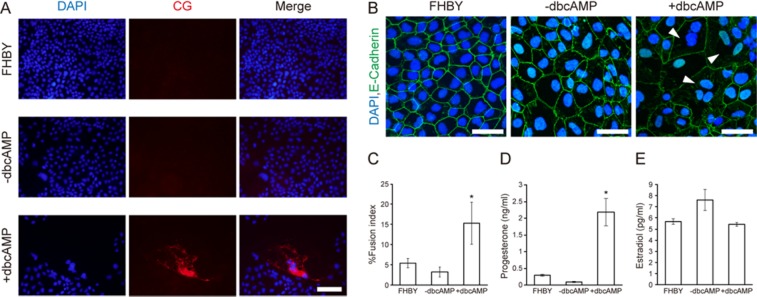
Differentiation of macTSC#2 towards multinucleated cells. (**A**) Fluorescent immunocytochemistry for CG protein. Scale bar = 100 µm. (**B**) Detection of multinucleated cells by immunofluorescence for E-cadherin and DAPI staining. Arrowheads show multinucleated (≥tri-n ucleated) cells. Scale bars = 40 µm. (**C**) The fusion index ± SD (%) calculated from four randomly chosen fields each of biological duplicates. Fusion index was calculated by (N-C)/T × 100 (N, nuclei in multinucleated cells; (**C**) number of multinucleated cells; T, total number of nuclei in a field of microscopic view). (**D**,**E**) Measurement of secreted steroid hormones. Progesterone (**D**) and Estradiol (**E**) in macTSC#2 culture medium were detected by ELISA (n = 3). *p < 0.05 vs. FHBY (two-tailed Student's *t*-test).

Human and non-human primate trophoblast cells differentiate to form multinucleated STBs, which secrete CG via cell fusion. Since results from qRT-PCR suggested that dbcAMP induced macTSC differentiation, we investigated whether macTSC#2 differentiates into STBs. The CG protein in dbcAMP-treated macTSC#2 was easily detectable by immunofluorescence (Fig. 5A). Furthermore, to detect the formation of multinucleated trophoblasts, cells were stained for E-Cadherin to visualize the cell border, and the frequency of appearance of multinucleated (≥ three nuclei per cell) cells was analyzed. While 5% and 3% of nuclei were fused to form multinucleated cells under FHBY and -dbcAMP conditions, respectively, dbcAMP treatment increased appearance frequency of such nuclei to 15% (Fig. 5B,C and Table S3). Macaque placenta secretes progesterone and estradiol during the gestation^33^. To see if differentiated macTSCs produce and secrete these steroid hormones, we measured the amount of progesterone and estradiol secreted in the culture media by ELISA (Fig. 5D,E). While there was no difference in secreted estradiol between FHBY and +dbcAMP conditions (Fig. 5E), the amount of progesterone was significantly increased in +dbcAMP condition (Fig. 5D). It is not known if there is no estrogen secretion, or the level was too low to detect over the background. These analyses suggested that at least some of the cells differentiated to the CG-positive, progesterone-producing STBs upon dbcAMP treatment of macTSC#2, but the differentiated STBs may not be fully functional.

### Differentiation of invasive EVT from macTSC

**Figure 6:**
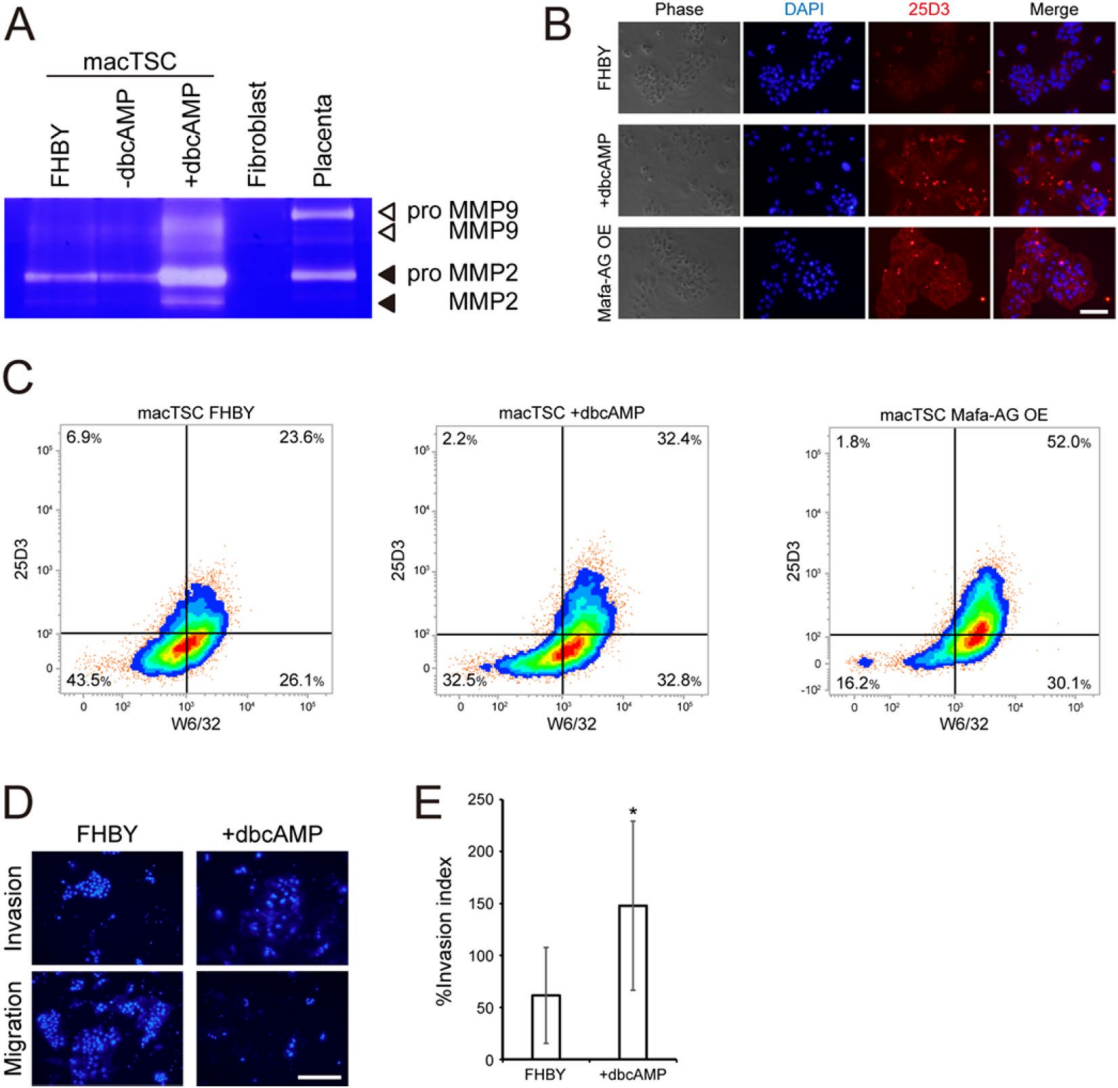
Differentiation of macTSC#2 toward invasive cells. macTSC#2 cells cultured for six days under the indicated conditions and were subjected to further analysis. (**A**) Detection of gelatinase activities in macTSC#2 by gelatin zymography. Fibroblast and placenta were used as negative and positive controls, respectively. (**B**) Fluorescent immunocytochemistry for Mafa-AG protein, a cynomolgus macaque homolog of HLA-G. Mafa-AG was detected by using the 25D3 antibody. A macTSC line overexpressing Mafa-AG protein (Mafa-AG OE) was used as a positive staining control. The high-intensity dots stained by 25D3 antibody were non-specific signals. Scale bar = 100 µm. (**C**) Flow cytometric analysis. PI staining-positive dead cells were gated out. The thresholds of positive and negative staining were decided using the isotype control antibody of Mafa-AG-overexpressing macTSC#2 (Fig. S5). (**D**) Cell invasion analysis of macTSC#2 cells. Cells were cultured on the FluoroBlok inserts with (Invasion) or without (Migration) Matrigel coating in the indicated conditions for four days. The nuclei of cells on the lower surface of the inserts were stained with Hoechst 33342 and photographed. The typical images are shown. Scale bar = 50 µm. (**E**) The invasion index (%) calculated from 835 and 364 total nuclei in FHBY and +dbcAMP conditions, respectively.

Human and non-human primate CTBs, especially those located in cell column, differentiate into another cell type including the invasive EVTs. To address whether macTSCs also differentiate towards EVT, we investigated the expression of EVT marker protein, and the invasiveness of macTSC. MMP2 and MMP9 reportedly degrade gelatin. We first performed gelatin zymography to detect MMP2 and MMP9 activity in the macTSC#2 cell extract, and the results show augmented level of MMP2 protein by the dbcAMP treatment (Fig. 6A). Similarly, the dbcAMP-treated macTSC#2 showed increased expression of Mafa-AG protein by immunofluorescence compared to cells in the FHBY condition (Fig. 6B).

The expression pattern of HLAs can be used to evaluate the subtype of human trophoblast cells^2^. While a lack of HLA-C ortholog, the only HLA type I molecule expressed in the human EVT, in the macaque genome hampers a straight application of this evaluation, cynomolgus STB and EVT were intensely stained by anti-human HLA type I antibodies^34,35^. However, undifferentiated CTB-like cells are detectable by the expression of HLA-A and -B ortholog. The flow cytometric analysis by using a pan anti-human HLA class I antibody, W6/32, and the 25D3 antibody for detecting Mafa-AG^35^, showed an increase in the ratio of HLA class I^+^/Mafa-AG^+^ putative EVT-like population from 23.6% to 32.4% by dbcAMP treatment at the expense of HLA class I^−^/Mafa-AG^−^ population (Fig. 6C, S5, and S6). This result suggested that some cells with EVT-like MHC expression profile existed even under the FHBY condition and that dbcAMP shifted the distribution of cell populations towards EVT-like cells.

Finally, we analyzed the invasiveness of macTSC#2 cells by employing the Matrigel invasion assay^36^. Cells were cultured for four days on the cell culture inserts, with or without Matrigel-coating, followed by counting the number of nuclei of invaded or migrated cells to calculate invasion-to-migration ratio (% invasion index). Although these analyses showed the presence of invasive EVT-like cells even in the FHBY condition in agreement with the flow cytometry results mentioned above, the ratio of invasive to migratory cells was increased significantly by the dbcAMP treatment (Fig. 6D,E). These results demonstrated that a part of macTSC#2 also acquired the invasive EVT-like phenotype upon dbcAMP treatment.

### Gene expression profile of macTSCs under the dbcAMP condition

**Figure 7:**
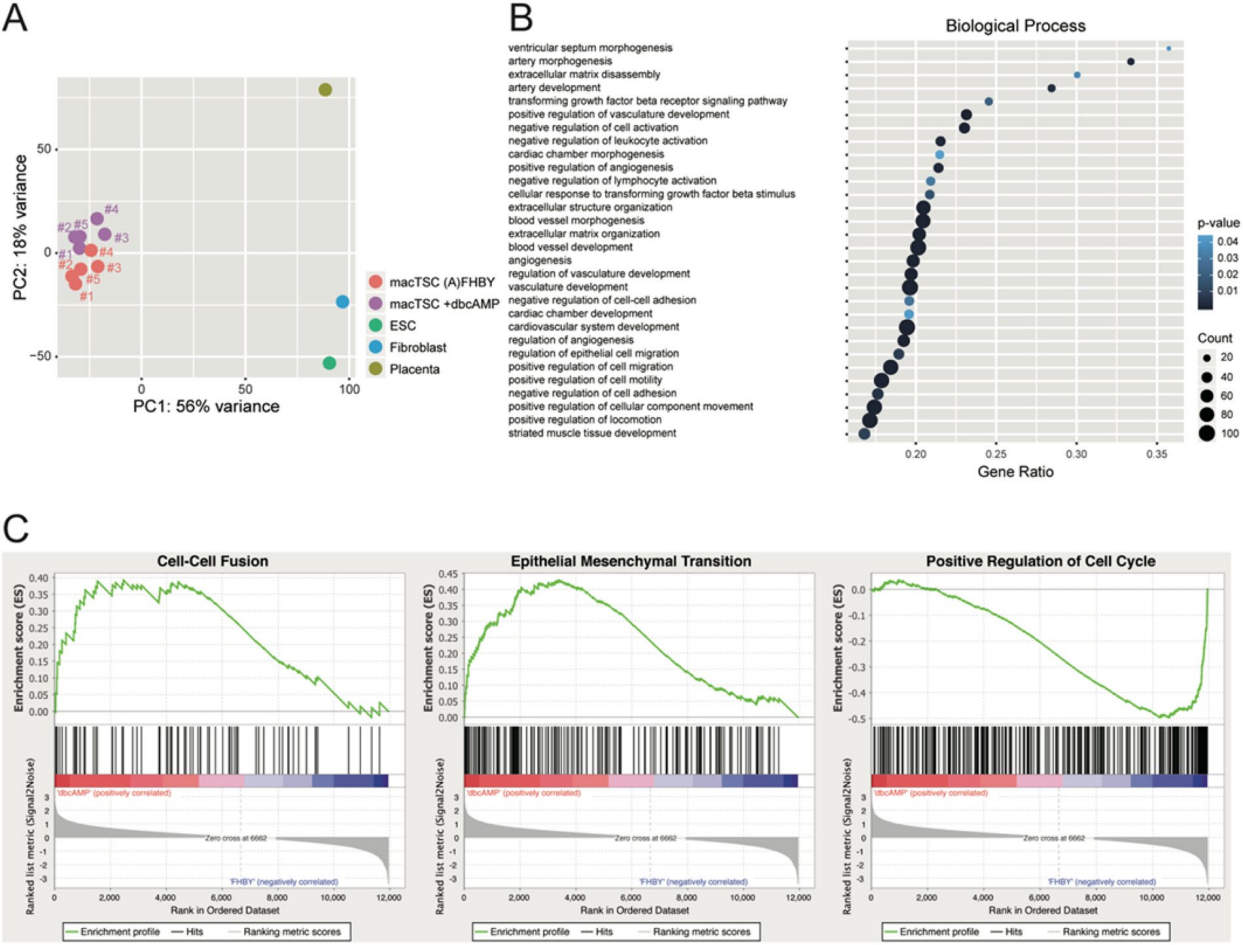
The effect of dbcAMP treatment for macTSCs. (**A**) PCA was performed using RNA-seq data of macTSCs in (**A**) FHBY and dbcAMP conditions, ESC, fibroblasts, and placenta. (**B**) Enriched GO terms associated with the genes >2-fold upregulated by the dbcAMP treatment. PANTHER (www.pantherdb.org) was used for GO analyses. Gene ratio, the ratio of the analyzed genes in the gene set of the GO term; Count, the number of analyzed genes in the GO term. *P*-values were calculated using Fisher's exact test and Bonferroni correction. (**C**) Gene set enrichment analysis (GSEA) was performed using gene expression data of RNA-seq by GSEA v4.0.3 Mac App from Broad Institute (software.broadinstitute.org/gsea/index.jsp).

The dbcAMP treatment induce human trophoblast differentiation toward multinucleated STB^8,30^. In this study, however, invasive EVT-like cells also appeared upon dbcAMP treatment of macTSC#2 (Figs. 5 and 6), suggesting that dbcAMP treatment also trigger EVT cell fate in macTSCs. To further resolve this question, we analyzed comprehensive gene expression profiles of macTSCs with dbcAMP by RNA-seq. From the result of PCA, the gene expression profiles of all five macTSC lines were slightly changed (Fig. 7A). The GO analysis of >2-fold upregulated genes after dbcAMP treatment in all macTSC lines showed enrichment of terms related to positive regulation of cell migration, vasculature development, and angiogenesis (Fig. 7B). Remarkably, these upregulated genes commonly included *GCM1*, *MMP2*, *CDH5*, and *HTRA4*, which are expressed exclusively or preferentially in the human placenta (Table S4)^37–39^. GO terms related to cell proliferation were enriched in >2-fold downregulated genes, (Figs. 7C and S7) reflecting the inhibition of cell growth by dbcAMP treatment. In addition, gene set enrichment analyses showed the upregulation of the genes associated with not only cell-cell fusion, but also epithelial to mesenchymal transition, the terms linked to multinucleated STB and invasive EVT formation, respectively (Fig. 7C). These results also suggested that all macTSC lines had the potential to differentiate towards both STB and EVT.

### Contribution of macTSC to the TE region of mouse blastocysts

**Figure 8:**
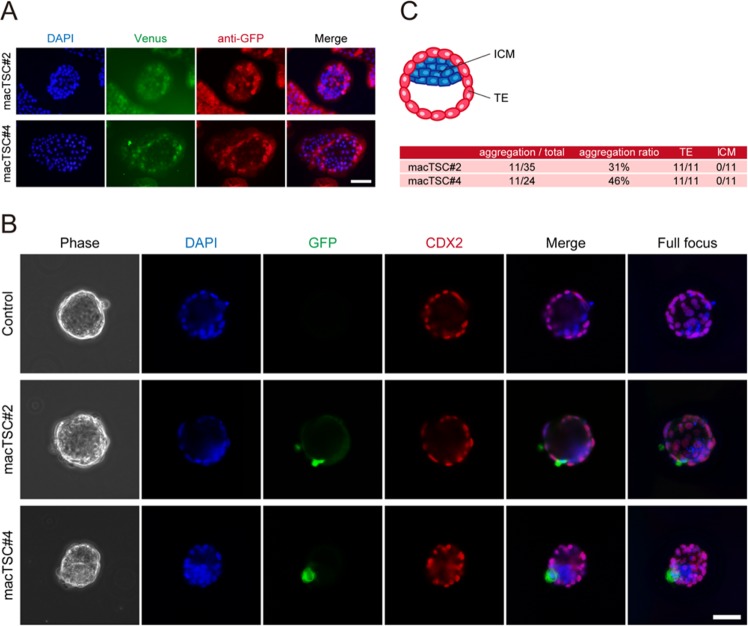
Contribution of macTSCs to the xenogeneic chimera blastocysts. (**A**)  Immunofluorescence images of Venus (modified GFP)-expressing macTSCs. Venus was detected by its fluorescence and by using anti-GFP antibody. Scale bar = 200 µm. (**B**) Images of xenogeneic chimera blastocysts generated by aggregating macTSC cells and mouse eight-cell embryos, at two days of culture. Scale bar = 50 µm. (**C**) Locations of Venus-positive cells in the xenogeneic chimera blastocysts.

Given that macTSCs were derived from blastocysts and showed the potential to differentiate toward both STB and EVT, we hypothesized that macTSCs will retain a TE cell-like feature at least partially. We addressed this question by performing a xenogeneic chimera assay by aggregating macTSC cells with mouse eight-cell stage embryos. For this purpose, several Venus-overexpressing macTSC lines from macTSC#2 and macTSC#4 were established to locate macTSC-derived cells in the xenogeneic chimeric blastocysts, and two of them, macTSC#2-Venus and macTSC#4-Venus, were used in the assay (Figs. 8A and S8). Notably, macTSCs were incorporated successfully into 31% (macTSC#2) and 46% (macTSC#4) of aggregates (Figs. 8B,C and S9). All macTSC cells were located at TE region, but not at ICM. These results demonstrated that macTSCs could participate in blastocyst formation by being a part of TE.

## Discussion

To date, human TSCs were not derived using similar or a variation of the conditions used for the derivation of mouse TSCs^14^. Moreover, no study investigated the effect of FGF4 on macaque trophoblast cells, underestimating the role of FGF4 in the proliferation of primate trophoblast stem/progenitor cells. In this study, we obtained a new type of cell lines, macTSC#1~#5, from cynomolgus monkey blastocysts in TS medium with FGF4, heparin, BMS493, and Y-27632. Although the exact origin of the cells is unknown, macTSCs met the recently proposed criteria for the definition of early human trophoblast cells^22^, such as expression of trophoblast/placenta-specific genes including the primate-specific C19MC miRNAs, and the hypomethylation of *ELF5* promoter (Figs. 2A, 3 and 6C). Previously identified T-E T-DMRs also showed DNA methylation status characteristic of trophoblast cell lineage (Fig. 2B). In addition, dbcAMP treatment not only enhanced the expression of STB and EVT marker genes/proteins, but also increased the formation of multinucleated cells and their invasiveness (Figs. 4, 5, and 6). Taken together, we conclude that the cell lines derived from cynomolgus monkey blastocysts, in this study, are essentially the trophoblastic cells with differentiation potential. The FGF4 signaling is essential for the maintenance of the undifferentiated state, and for promoting the proliferation of mouse TSCs^13,40^. FGF4 also enhanced proliferation of macTSCs in this study, although it appeared dispensable for the maintenance of macTSCs’ undifferentiated state (Fig. 1C,D). Thus, the macTSCs are the first primate trophoblast stem cell lines whose proliferation is promoted by FGF4.

It should be noted that we cannot rule out the possibility that the macTSC line is a heterogeneous cell population, including progenitors of both STB and EVT, and that the dbcAMP treatment may have induced differentiation of both these progenitor cells. In the flow cytometric analysis, around 30% of cells were pan HLA class I^+^/Mafa-AG^−^ in any conditions (Fig. 6C). Although the identity of this population is ambiguous, freshly isolated cynomolgus monkey trophoblast showed higher intensity in W6/32 staining compared with isotype control^35^. Besides, W6/32 and 25D3 antibodies recognized STB in chorionic villi of cynomolgus monkey placenta^35^. Thus, pan HLA class I^+^/Mafa-AG^−^ population may be composed of CTB- and STB-like cells, and pan HLA class I^+^/Mafa-AG^+^ population might include not only EVT- but also a few STB-like cells. Another concern regarding the heterogeneity is a possible contamination of ICM-derived cells. We would like to point out that the cell lines were established in three different initial culture conditions. One of the three conditions did not even include any exogenous factors (Fig. 1A). If there were any ICM-derived cells survived, they likely differentiated into the cells with different phenotypes depending on the culture conditions. However, the five cell lines showed highly similar gene expression profiles to each other (Fig. 4A), implying that there is no or minimal contamination of ICM-derived cells in the established cell lines. A single-cell RNA-sequencing or a single-cell cloning and subsequent differentiation anlyses can conclude if macTSCs are indeed multipotential stem cells. This caveat will be addressed in a future study.

RNA-seq analyses of macTSCs revealed dbcAMP-dependent upregulation of *CD9* (Table S2), which is proposed as a marker for putative human EVT progenitor cells^41^. This might suggest that macTSCs are in a developmentally immature, and an undifferentiated state compared to EVT progenitor cells, which are known as cell column trophoblast located in the tip of villi, and that dbcAMP induced differentiation of a part of macTSCs towards EVT through a temporal EVT progenitor state. RNA-seq and subsequent GO term enrichment analyses showed that genes associated with the terms related to the cardiovascular system, such as “ventricular septum morphogenesis,” and “artery morphogenesis,” were upregulated upon dbcAMP treatment (Fig. 7B). A recent finding showing placental defects are highly prevalent in mutant mice with embryonic abnormalities in heart, brain, and vascular system may partly explain our GO term enrichment results^42^. In other words, genes with relevant functions in trophoblast or placenta are likely associated with the terms related to the cardiovascular system. We, therefore, consider that the GO analysis result is not conflicting with the feature of trophoblast cells.

Vandevoort *et al*. have previously reported the derivation of TSC lines from TE of rhesus monkey blastocysts, plated on collagen-coated dishes after initial expansion with rhesus embryonic feeder cells, but without any exogenous factors^18^. While these cells expressed trophoblast marker genes such as KRT7 and CD9, CDX2 expression was undetectable by both immunohistochemistry and RT-PCR. In addition, the expression of CGB, one of the STB markers, was observed without induction of differentiation, suggesting that the rhesus TE-derived cells are actually in a more differentiated state than our macTSCs. It was noted that the expression of CDX2 increased in the rhesus TE-derived cells when cultured in the presence of Activin A^19^. In contrast, there was no expression of CDX2 in macTSCs (Fig. S10), even in the presence of Activin A. Furthermore, the effect of FGF4 on the rhesus monkey TSCs was not investigated.

Recently, human TSCs (hTSCs) were established from blastocysts and early gestation placentae^15^. Both hTSCs and macTSCs expressed CTB marker genes, such as *TEAD4*, *TP63*, and *FZD5* in the undifferentiated state, while upregulation of STB and EVT markers, including *CGA*, *CGB*, *MMP2*, *ITGA5*, and *CD9* was induced by the differentiation condition (Figs. 5, 6 and Table S2). Thus, hTSCs and macTSCs show similarities in the expression pattern of each of the trophoblast marker. In addition, both macTSCs and hTSCs have differentiation capacity toward STBs and EVTs. However, the requirement of exogenous factors is significantly different. While the EGF and Wnt signaling activator were essential for the maintenance of hTSCs and CTBs^15,43^, PD153035, an EGF inhibitor, caused slight reduction without complete inhibition of the proliferation of macTSCs in the presence of FGF4 (Fig. S11). Thereby, demonstrating that the effect of EGF-like activities in the FBS, if present, on the proliferation of macTSCs is negligibly weak. FGF4 was not essential to maintain human TSCs, whereas it enhanced the proliferation of macTSCs. Both human and macaque have the discoidal hemochorial placenta that comprised mostly of CTB, STB, EVT, and extraembryonic mesodermal cells^16,44^. In humans, after the attachment of the blastocyst on the uterine wall, trophoblasts invade decidua to lead the whole embryo to embed within endometrium^16^. In the macaque, the blastocyst does not invade decidua and stays at the surface of the uterine wall resulting in the formation of a secondary placenta on the opposite side of the primary implantation site^45^. The observed difference in the requirement of growth factors between hTSCs and macTSCs could be due to a difference in the placentation process described above. Induction of macTSCs’ differentiation toward STB by dbcAMP treatment was seemingly inefficient compared to that of hTSCs^15^, regardless of statistically significant increase in the marker gene expression and the fusion index. Considering this argument along with the fact that macTSCs have a relatively unstable karyotype, the FHBY condition may be suboptimal. It would be worthwhile to investigate similarities in transcriptome and epigenome of the hTSCs and macTSCs, furthermore, how macTSCs may perform when grown in hTSCs culture conditions should be investigated in future studies.

In mice, TSC lines demonstrated relevant roles of certain transcription factors (TFs), such as Cdx2, Elf5, and Eomes^46^, in the specification, maintenance, and differentiation of TSCs. However, specific roles of these TFs in the primate trophoblast lineage remain obscure, mostly due to lack of appropriate model systems, as recently discussed by Knöfler *et al*.^47^. As these authors suggested, the availability of the recently developed hTSCs^15^, and 3D placental organoids^43,48^ will accelerate the functional analysis of such TFs. Thereby, deepening our knowledge about TSCs and key TFs involved in trophoblast lineage. The establishment of macTSC lines, as in the present study, can facilitate implementation of such research experiments. Knowing more molecularly-specific similarities and differences between the trophoblast of human and non-human primates is a prerequisite for pre-clinical studies. The macTSCs provide a useful tool for revealing key TF networks that are common to the primate trophoblast, and for delineating human-specific and macaque-specific functions of underlying TFs. In addition, these non-human primate TSCs will provide cellular proxy for *in vivo* analyses, such as the chimeric analysis, which are prohibited in humans for ethical reasons. Such attempt may reveal whether macTSCs show the TE-like feature and contribute to the placenta in a future study.

## Methods

### Reagents

All reagents were purchased from Wako (Osaka, Japan) unless otherwise noted. All PCR primers were purchased from Sigma-Aldrich (Tokyo, Japan) or Eurofins Genomics (Tokyo, Japan).

### Experimental animals

Cynomolgus monkey embryos were produced by IVF and cultured in the Department of Stem Cells and Human Disease Models, Research Center for Animal Life Science, Shiga University of Medical Science. CD-1 (ICR) mice were purchased from Oriental Yeast Co Ltd. (Tokyo, Japan). All animal experiments were conducted following the guidelines approved by the Institutional Animal Care and Use Committee of the Graduate School of Agricultural and Life Sciences, the University of Tokyo (approval ID: P15-89, P17-159), and by the Shiga University of Medical Science (2015-5-11).

### Establishment of trophoblastic cell lines from cynomolgus monkey blastocysts

Cynomolgus monkey blastocysts (five embryos in total) were transferred to the University of Tokyo, and cultured for 2-8 days with or without mitomycin C-treated mouse embryonic fibroblasts (MEF) in basal culture medium (TS medium) consisting of RPMI-1640 medium supplemented with 20% fetal bovine serum (FBS, Biowest, Nuaillé, France), 2 mM L-glutamine, 1 mM sodium pyruvate, and 0.1 mM 2-mercaptoethanol, and exogenous reagents as indicated in Fig. 1A. The reagents used were 10 ng/ml Activin A (R&D Systems, Minneapolis, US), 25 ng/ml FGF4, 1 µg/ml Heparin (Sigma-Aldrich), 1 µM BMS493 (TOCRIS, Bristol, UK), and 10 µM Y-27632. The cells were incubated at 37 °C in a humidified atmosphere of 5% CO_2_, and the culture medium changed once every two days after the blastocysts firmly attached to the dish. When blastocyst outgrowth expanded, it was trypsinized and re-plated on a larger multi-well plate or dish.

### Cell culture and induction of differentiation

macTSCs were routinely cultured in the TS medium supplemented with FGF4, heparin, BMS493, and Y-27632 (FHBY) to maintain the proliferative state with media change every two days. To induce differentiation, macTSCs were exposed to 1 mM dbcAMP (Sigma-Aldrich) for 6 days in the absence of FHBY (+dbcAMP condition).

### Cell growth assay

Cells were seeded at 1 × 10^5^ per well in 6-well plates and pre-cultured for one day in the AFHBY condition (see Fig. 1A for the abbreviation). Then, cells were re-fed with fresh TS medium supplemented with a different combination of factors. The cells were harvested at 0, 2, and 4 days after changing the culture conditions and counted by the Countess™ automated cell counter (Invitrogen, Carlsbad, US).

### Chromosome count

To pause cell cycle at metaphase, the KaryoMAX^®^ Colcemid™ Solution (Gibco, Dublin, Ireland) was added to the culture medium at 1:100 dilution and the cells incubated at 37 °C for 24 h. The cells were then trypsinized and centrifuged. After discarding the supernatant, the pellet was re-suspended in 0.06 M KCl/citrate-saline solution and incubated at 37 °C for 20 minutes. Cells were fixed in methanol/acetic acid (3:1) twice. The cell suspension was placed on a glass slide and stained by DAPI (DOJINDO, Kumamoto, Japan), and the number of chromosomes were counted in the fluorescent images.

### Bisulfite sequencing analysis

The cytosine-to-uracil conversion of extracted genomic DNA was by using the EZ DNA Methylation-Direct™ Kit (Zymo Research, Irvine, US). Then, the treated genomic DNA amplified with Bio Taq HS DNA polymerase (BIOLINE Meridian Bioscience, Cincinnati, US) with the specific primer sets for target regions (Table S5). After electrophoresis, PCR products were purified by Wizard^®^ SV Gel and PCR Clean-Up System (Promega, Madison, US), cloned into pGEM-T Easy Vector (Promega), and sequened using BigDye Terminator^®^ v.31 (Applied Biosystems, Foster City, US). The sequence analyses and statistical comparisons (nonparametric two-tailed Mann-Whitney U-test) were by QUMA web service (http://quma.cdb.riken.jp/).

### RNA-Sequence analysis (RNA-seq)

Total RNAs of macTSC lines (#1 and #2 in AFHBY condition, #3-5 in FHBY condition), fibroblast, and the day 50 placenta were extracted using TRIzol (Life Technologies, Carlsbad, US) according to manufacturer's instruction. RNA-seq was done at Takara Bio Inc. (Kusatsu, Japan). In brief, cDNA was synthesized using oligo(dT) primer and SuperScript III Reverse Transcriptase (Thermo Fisher Scientific, Waltham, US). Prepared libraries were sequenced with HiSeq (Illumina, San Diego, US). Upon receipt of the RNA-seq data, reads were mapped to the version 5.0 assembly (Ensembl) of the *Macaca fascicularis* genome using HISAT2 v2.0.1-beta, by using a transcript assembly to guide mapping. Putative transcripts were identified in macTSC samples, ESC, fibroblast, and placental samples using Stringtie v 1.2.2. Stringtie was then used to merge the nine transcript sets, guided by the Cynomolgus genome annotation set (CY.v6) to generate a transcript model to be u sed in further analysis. Reads from all samples were assigned to genes in the assembled genome model using FeatureCounts v.1.4.6, and the count table loaded into DESeq2 for differential expression analysis. The raw and processed data are available at the GEO database (GSE131795).

### Clustering analysis

Pearson correlation coefficient was calculated using RNA-seq data of macTSCs ((A)FHBY), ESC, fibroblast, and placenta by R (https://www.r-project.org/). The clustering was generated by the Euclidean distance and farthest neighbor method.

### Gene ontology (GO) analysis

GO analysis was performed using RNA-seq data of macTSCs (FHBY and dbcAMP), and ESC by PANTHER (www.pantherdb.org). Fisher's exact test and Bonferroni correction were used to calculate the *P*-values.

### qRT-PCR for miRNAs

Total RNA of cynomolgus monkey somatic tissues for miRNA analysis was extracted from brain, kidney, lung and small intestine at Kyoto University. cDNA was synthesized from total RNA by Mir-X miRNA First-Strand Synthesis Kit (Clontech, Mountain View, US) and amplified using miR-specific primers shown in Table S5. The expression level was normalized to that of U6 snRNA.

### qRT-PCR

cDNA was synthesized using SuperScript^®^ III Reverse Transcription (Invitrogen). qRT-PCR was performed using THUNDERBIRD™ SYBR^®^ qPCR Mix (TOYOBO, Osaka, Japan). Amplification and fluorescence detection were carried out using Light Cycler^®^ 96 (Roche, Basel, Switzerland). Primers used are shown in Table S5.

### Fluorescence immunocytochemistry

Cells grown on coverslips were fixed with 4% paraformaldehyde in PBS(-). Fixed cells were then permeabilized with 0.1% Triton-X in PBS(-) and incubated with 5% BSA, 0.1% Tween-20 in PBS(-) overnight. Subsequently, the cells were incubated with primary antibodies at 4 °C overnight. Primary antibodies used in this study are listed in Table S6. The signals were visualized by Alexa Fluor^®^ 488 goat anti-mouse IgG (A11029, Invitrogen) or Alexa Fluor^®^ 594 goat anti-rabbit IgG (A11037, Invitrogen). After nuclear counter-staining with DAPI (DOJINDO), the cells were mounted on glass slides with VECTASHIELD^®^ H-1000 (Vector Laboratories, Burlingame, US). The cells containing three or more nuclei were defined as the multinucleated cells in this study. The fusion index (%) was calculated by the following formula: [(the number of nuclei in the multinucleated cells in a field of microscopic view) – (the number of multinucleated cells in a field of microscopic view)]/(total number of nuclei in a field of microscopic view)] × 100 (Table S3). 25D3 antibody, which has been shown to detect Mafa-AG, a cynomolgus monkey homolog of human HLA-G^35,49^, was kindly provided by Dr. Thaddeus G. Golos, University of Wisconsin-Madison.

### Measurement of steroid hormones in culture medium by ELISA

After the 6-days differentiation of macTSC#2, culture medium was replaced to a fresh TS medium containing 0.1% FBS and further cultured for 9 hours. The culture medium was then collected, centrifuged to remove the dead cells and debris, and the supernatant was used for ELISA. One ml of culture medium was extracted twice with 2 ml of diethyl ether and incubated at −80 °C for 20–30 minutes. The supernatant was transferred to a new glass tube and dried up at 55 °C. The extract was diluted with 200 µl of ELISA Buffer in Progesterone ELISA Kit (Cayman Chemical, Ann Arbor, US) or Assay Buffer in DetectX^®^ SERUM 17β-ESTRADIOL Enzyme Immunoassay Kit (Arbor Assays, Ann Arbor, US). The measurement of progesterone and estradiol was performed by Progesterone ELISA Kit and DetectX^®^ SERUM 17β-ESTRADIOL Enzyme Immunoassay Kit following manufacturers’ instructions.

### Protein extraction and zymography

Total protein was extracted with RIPA buffer containing 1 mM PMSF. Total proteins (5 µg) were then separated by SDS-PAGE using 10% acrylamide gel containing 1.0 mg/ml gelatin. After the electrophoresis, the gel was incubated twice in the renaturation solution (2.5% Triton X-100) for 40 minutes each followed by overnight incubation in developing buffer (50 mM Tris-HCl pH 7.4, 200 mM NaCl, 5 mM CaCl_2_, and 1% Triton X-100) at 37 °C. After incubation, the gel was stained with Coomassie blue stain and de-stained using 30% methanol/10% acetic acid solution until the bands became visible.

### Establishment of Mafa-AG/Venus-overexpressing macTSC lines

A macTSC#2 line overexpressing Mafa-AG was established to set the threshold in flow cytometric analysis. A full-length ORF of *Mafa-AG* was amplified by using a primer set that added a 3xFlag tag at the N-terminus (Table S5) and cloned into pENTR™/D-TOPO^®^ (Invitrogen). The 3xFlag-Mafa-AG cDNA insert was transferred into a destination vector, including Venus and puromycin-resistant genes^50^ using Gateway^®^ LR Clonase™ II (Invitrogen). The resulting Mafa-AG/Venus-expression vector was transfected into macTSC by using jetPRIME® *in vitro* DNA & siRNA Transfection Reagent (Polyplus Transfection, Illkirch, France) for 6 hours in the FHBY condition. Transfected cells were selected with 10 µg/ml puromycin for 48 hours after the transfection. Single Venus-positive colonies were picked and expanded separately in the FHBY condition. A cell line with a uniform and strong Venus fluorescence was used in the present study.

### Flow cytometry

Cells were harvested with trypsin, suspended in 1% BSA/PBS(-) and incubated at room temperature for 30 min. Then, 10 µg of the primary antibodies were added, followed by another incubation for 30 min at room temperature. Refer to Table S6 for the primary and secondary antibodies used in this assay. The dead cells were stained with propidium iodide (Sigma-Aldrich). The expression of Mafa-AG and HLA class I was analyzed by FACSVerse™ Flow Cytometer. Isotype controls of anti-Mafa-AG and anti-HLA class I antibody was used to draw a threshold line (Fig. S5).

### Invasion assay

macTSC#2 cells were pre-cultured in the proliferative (FHBY) or differentiation (+dbcAMP) conditions. macTSC#2 cells in the proliferative or differentiated state were cultured for four days in FluoroBlok™ cell culture inserts with 8.0 µm pore size (Corning, New York, US) covered with Matrigel^®^ matrix (Corning) in FHBY or +dbcAMP conditions. Then, 1.5 × 10^3^ (FHBY) or 1.0 × 10^4^ (+dbcAMP) cells were placed on the insert. The inserts were filled with the TS medium supplemented with 0.1% FBS so that the cells get attracted to the TS medium supplemented with 20% FBS in the bottom wells. Cell nuclei on the lower surface of the inserts were stained with Hoechst 33342 (Lonza, Basel, Switzerland) and photographed. Migration assay, without Matrigel coating, was also performed in parallel as a control. The invasion indices, [(number of invaded cells/number of migrated cells) × 100] (%), were calculated for each condition from 6 (FHBY) and 21 (+dbcAMP) randomly chosen microscopic images each of biological duplicate (835 and 364 total nuclei in FHBY and +dbcAMP conditions, respectively). Since, dbcAMP treatment severely block cell proliferation, more images were required to achieve enough number of nuclei.

### Establishment of Venus-overexpressing macTSC lines

macTSC#2 and #4 lines overexpressing Venus was established for xenogeneic chimera assay. The 3x Flag Venus-expression vector^50^ was transfected into macTSC using jetPRIME® *in vitro* DNA & siRNA transfection reagent (Polyplus Transfection, Illkirch, France) for six hours in the FHBY condition. Transfected cells were selected with 10 µg/ml puromycin for 48 hours after the transfection. Single Venus-positive colonies were selected and expanded separately in the FHBY condition.

### Western blotting

Total protein was extracted with RIPA buffer containing 1 mM PMSF. Total proteins (5 µg) were separated by SDS-PAGE and transferred onto Immobilon PVDF membrane (Millipore, Darmstadt, Germany). The membrane was incubated in blocking buffer (5% BSA, 0.1% Tween-20/TBS) for 1 hour, followed by overnight incubation with anti-GFP antibody or IgG (Table S6) at 4 °C. After rinsing with wash buffer (0.05% Tween-20/TBS), the membrane was incubated with the secondary antibody diluted in blocking buffer at room temperature for 1 hour. The protein of interest was detected using ImmunoStar Basic.

### Xenogeneic chimera assay

Chimeric embryos were generated following the standard protocol^51,52^. In brief, mice 8-cell embryos were flushed from the oviduct and rinsed several times with M2 medium. Denuded embryos were co-cultured in KSOM medium with a couple of macTSC-Venus cells for 48 h to form a blastocyst. Blastocysts were fixed with 4% PFA, 1% PVA in PBS(-), rinsed in wash buffer (1% PVA in PBS(-)), and transferred to permeabilization buffer (0.1% Triton X-100, 1% PVA in PBS(-)). After permeabilization, the embryos were incubated in blocking buffer (5% BSA, 0.1% Tween-20 in PBS(-)) at 4 °C overnight. Subsequently, anti-GFP pAb or anti-CDX-2 (Table S6) antibodies were added before another incubation at 4 °C overnight. Alexa Fluor^®^ 488 goat anti-rabbit IgG or Alexa Fluor 594-AffiniPure Goat Anti-Mouse IgG (H+L) (Table S6) were used as a secondary antibody. After nuclear staining with DAPI (DOJINDO), the blastocysts were mounted on slide glasses with VECTASHIELD^®^ H-1000 (Vector Laboratories) for fluorescent microscopy.

### Principal component analysis (PCA)

PCA was performed by R (https://www.r-project.org/) using the RNA-seq data of macTSC#1-5, ESC, fibroblast, and placental samples.

### Gene set enrichment analysis (GSEA)

GSEA was performed using RNA-seq data of macTSCs by GSEA v4.0.3 Mac App from Broad Institute (software.broadinstitute.org/gsea/index.jsp). The gene set of cell-cell fusion (GO:0140253) was created using AmiGO 2 (amigo.geneontology.org/amigo).

### Statistical analyses

The Tukey-Kramer test was performed for Figs. 1D, 3, S1C, and S11. A two-tailed Student's *t*-test was performed for Figs. 4C–E, 5C–E, 6E, and S1E. *P*-values of GO analyses (Figs. 4B, 7B, S4, and S7) were calculated using Fisher's exact test and Bonferroni correction. The difference in the DNA methylation status (Fig. 2A,B) was detected using nonparametric two-tailed Mann-Whitney U-test. For the Tukey-Kramer test, the different letters show a significant difference (*P*-value < 0.05). For other analyses, the statistically significant differences were represented by *(*P*-value < 0.05).

### Accession codes

GSE131795.

## Supplementary information

Supplementary information.
