# Supplementary material for: Establishment of macaque trophoblast stem cell lines derived from cynomolgus monkey blastocysts
Source: Sci Rep. 2020 Apr 22;10:6827. doi: 10.1038/s41598-020-63602-7 (PMC7176671; doi:10.1038/s41598-020-63602-7)
Supplement: Supplementary file 1 — Supplementary information. [file 41598_2020_63602_MOESM1_ESM.docx]

**Supplementary information**

**TITLE**

Establishment of macaque trophoblast stem cell lines derived from cynomolgus monkey blastocysts.

**AUTHORS**

Shoma Matsumoto^1^, Christopher J. Porter^2^, Naomi Ogasawara^1^, Chizuru Iwatani^3^, Hideaki Tsuchiya^3^, Yasunari Seita^3^, Yu-Wei Chang^4^, Ikuhiro Okamoto^5, 6, 7^, Mitinori Saitou^5, 7, 8^, Masatsugu Ema^3, 7^, Theodore J. Perkins^2^, William L. Stanford^2, 9^, and Satoshi Tanaka^1, *^

^1^ Department of Animal Resource Sciences, Graduate School of Agricultural and Life Sciences, The University of Tokyo, Tokyo 113-8657, Japan;

^2^ The Ottawa Hospital Research Institute, Ottawa ON K1H 8L6, Canada;

^3^ Department of Stem Cells and Human Disease Models, Research Center for Animal Life Sciences, Shiga University of Medical Sciences, Shiga 520-2192, Japan;

^4^ Department of Veterinary Physiology, Graduate School of Agricultural and Life Sciences, The University of Tokyo, Tokyo 113-8657, Japan;

^5^ Department of Anatomy and Cell Biology, Graduate School of Medicine, Kyoto University, Kyoto 606-8501, Japan;

^6^ Japan Science and Technology (JST), Exploratory Research for Advanced Technology (ERATO), Kyoto, Japan;

^7^ Institute for Advanced Study of Human Biology (WPI-ASHBi), Kyoto University, Kyoto 606-8501, Japan;

^8^ Center for iPS Cell Research and Application (CiRA), Kyoto 606-8507, Japan;

^9^ Department of Cellular and Molecular Medicine, University of Ottawa, Ottawa K1H 8M5, Canada;

^*^ To whom correspondence should be addressed: Satoshi Tanaka.

Department of Animal Resource Sciences, Graduate School of Agricultural and Life Sciences, The University of Tokyo, Tokyo 113-8657, Japan

TEL/FAX: +81-3-5841-5472/+81-3-5841-8189

Email: asatoshi@mail.ecc.u-tokyo.ac.jp

**Table S1-S6**

**Figure S1-S12**

**Supplementary Table S1.** Top 30 list of > 2-fold over-represented genes in RNA-seq data of all macTSCs* compared to that of ESC.


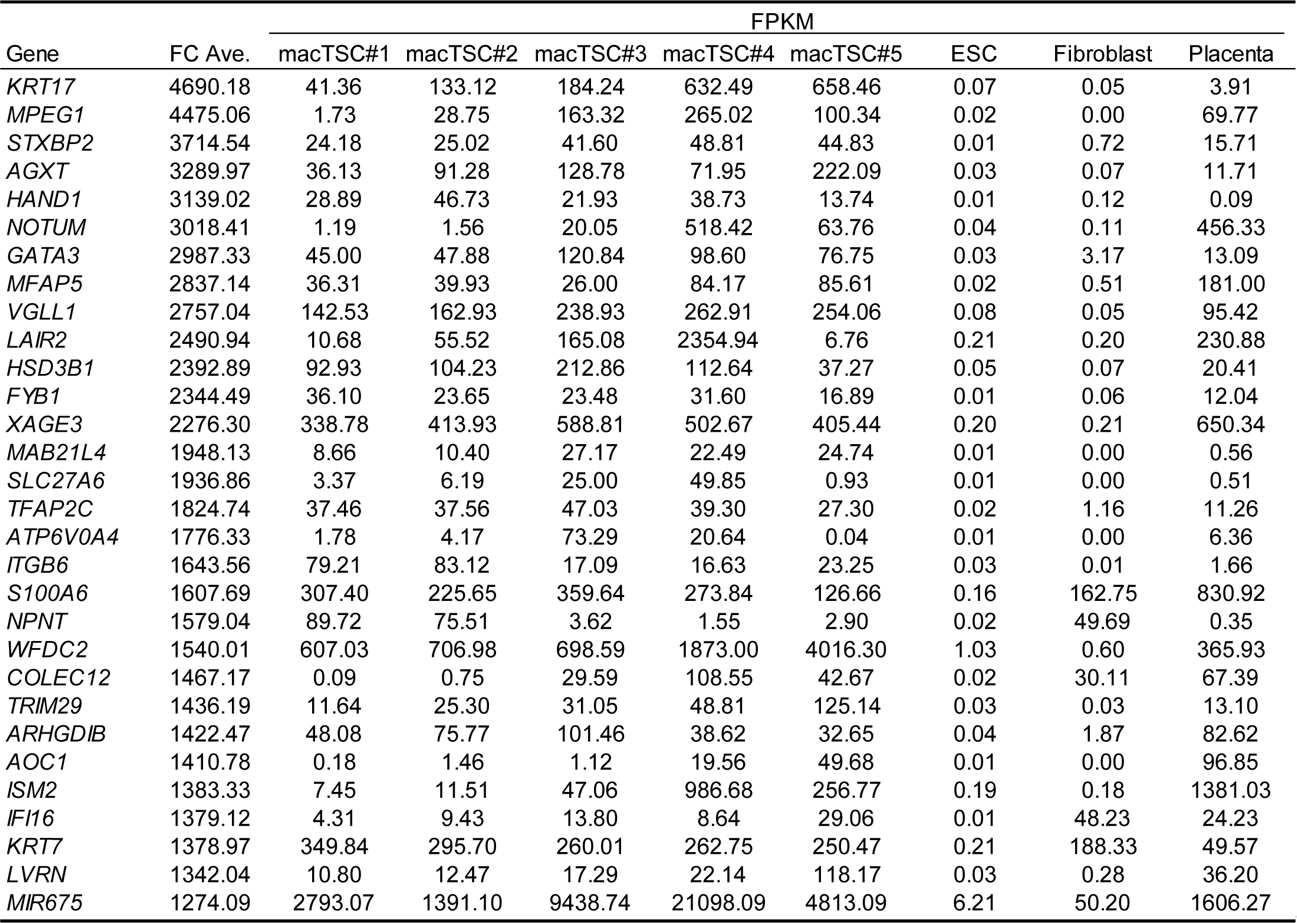


*, in AFHBY (#1, 2) or FHBY (#3-5) conditions; FC, fold-change

**Supplementary Table S2.** FPKM value of trophoblast markers from RNA-seq data.


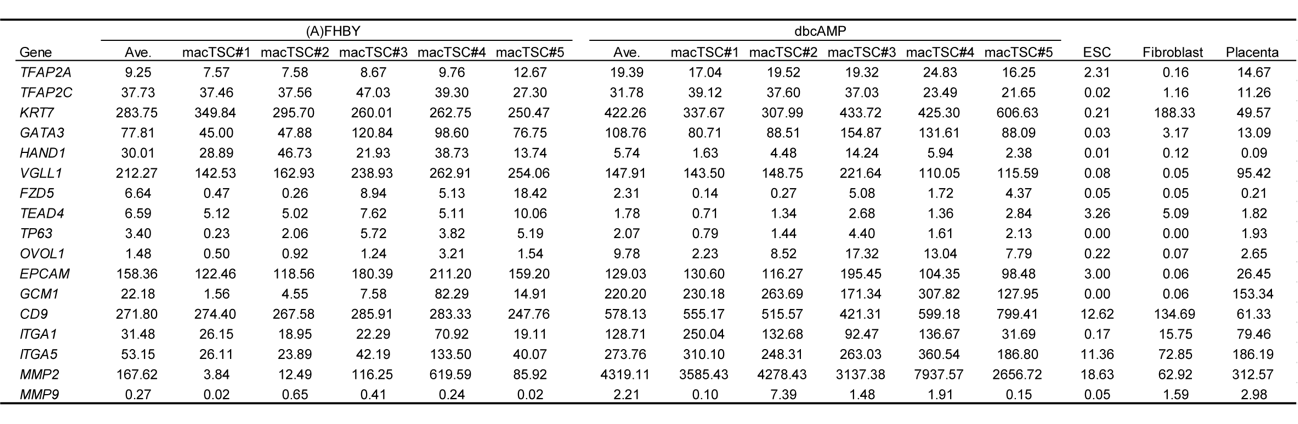


**Supplementary Table S3.** The number of single and multinucleated (≥ 3 nuclei) cells.


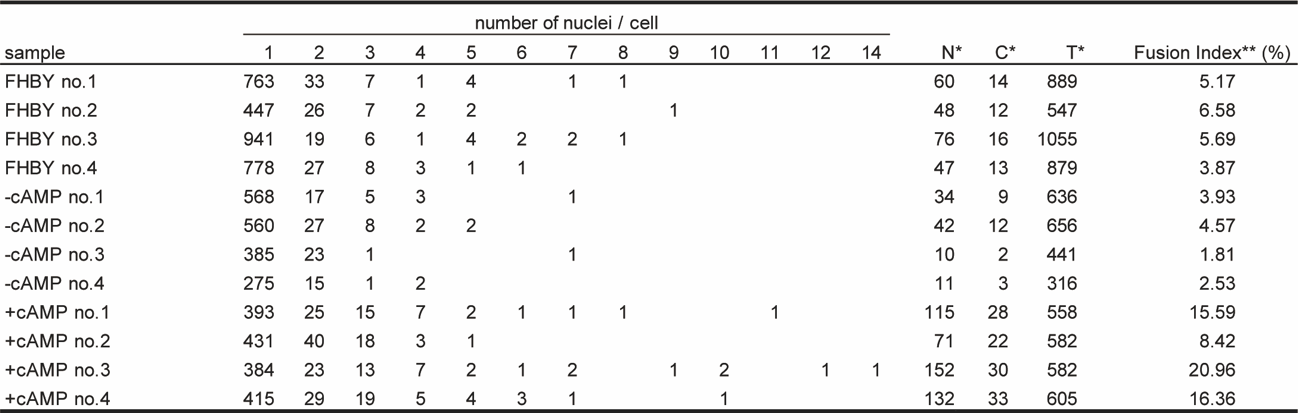


* N, the number of nuclei in the multinucleated cells; C, the number of multinucleated cells; T, the total number of nuclei.

** Fusion index = (N-C)/T

**Supplementary Table S4.** Top 30 list of 2-fold up-regulated genes by dbcAMP treatment in macTSCs.


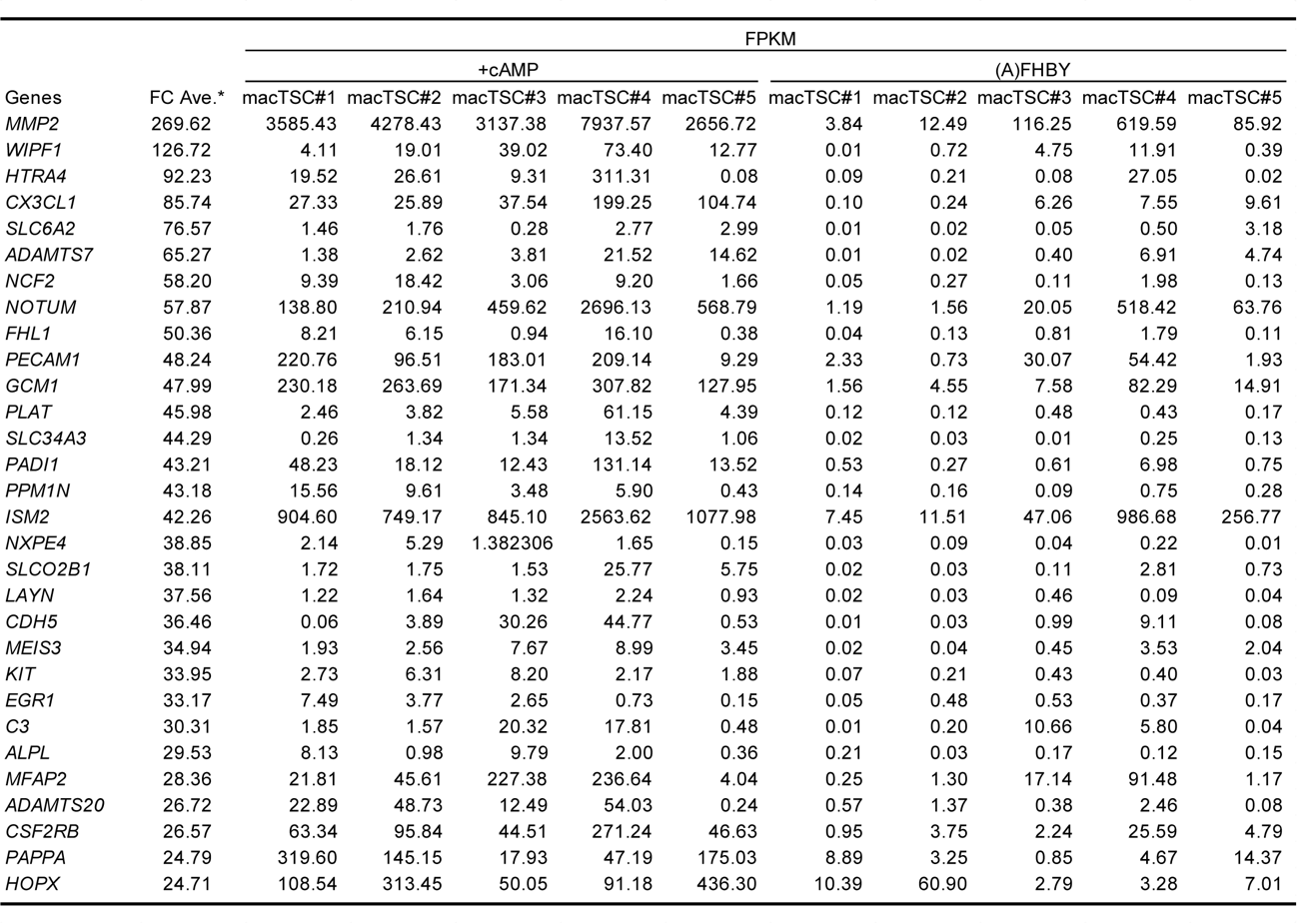


*, +cAMP vs AFHBY (#1, 2) or FHBY (#3-5); FC, fold-change

**Supplementary Table S5.** Primers list.


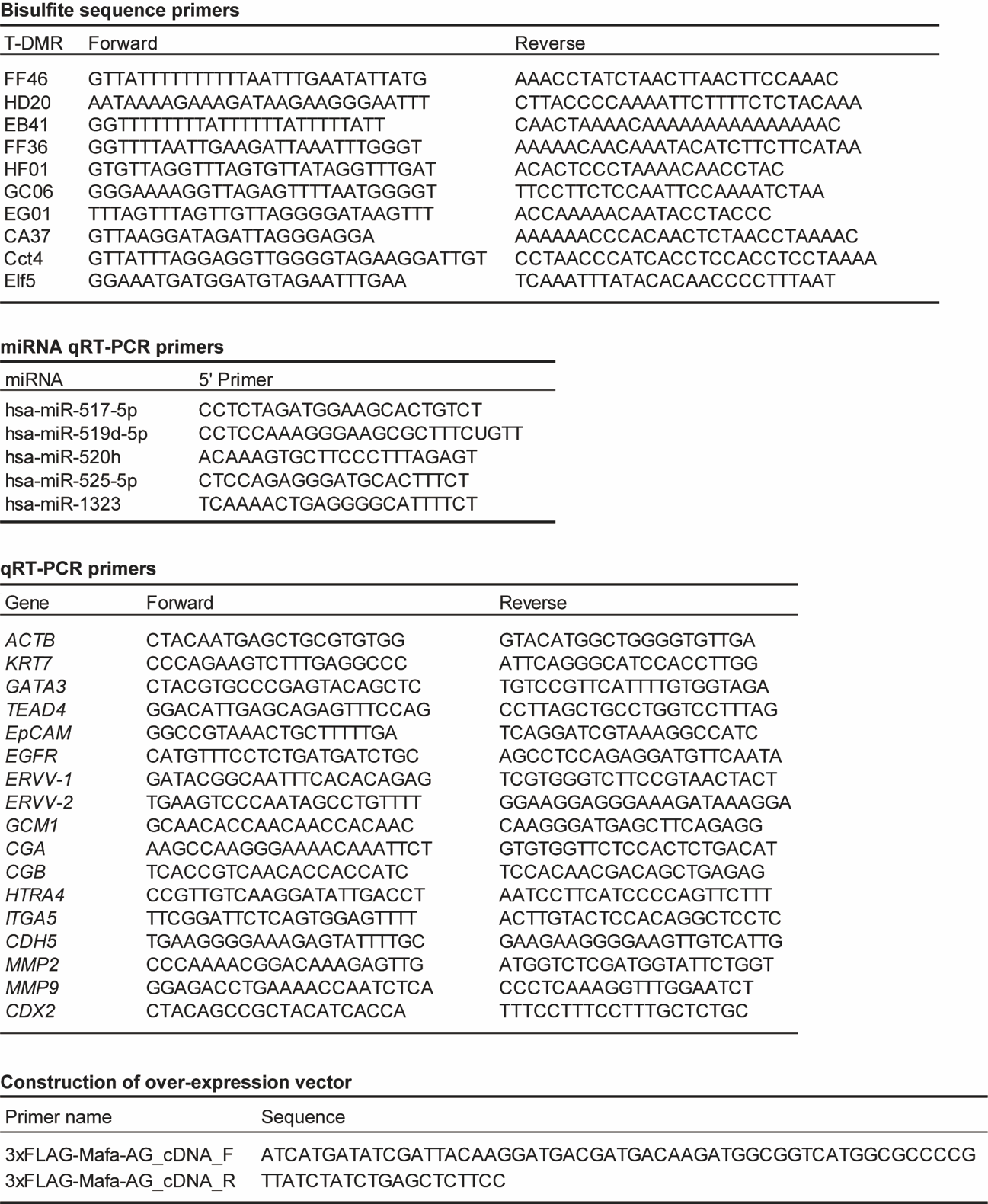


**Supplementary Table S6.** Antibody list.


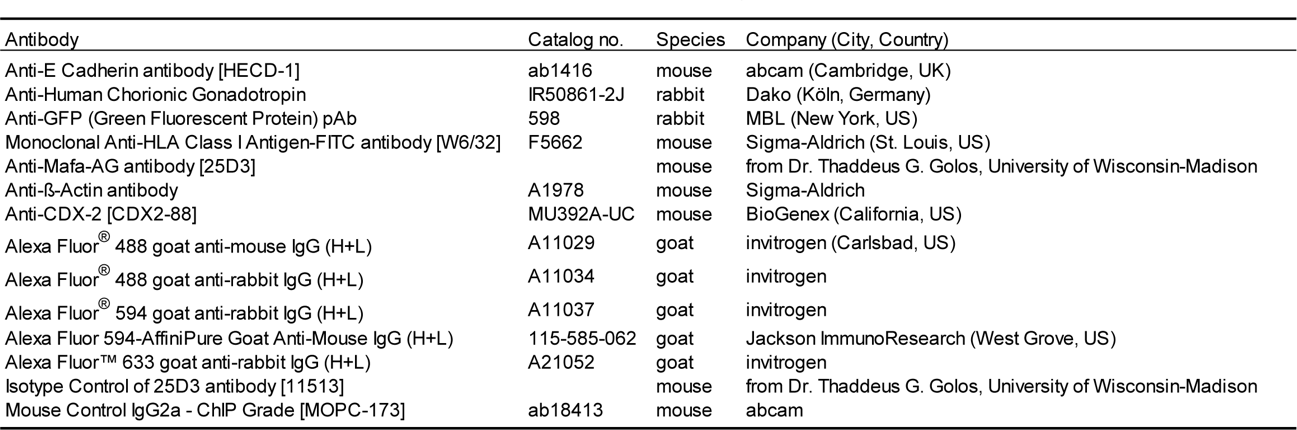


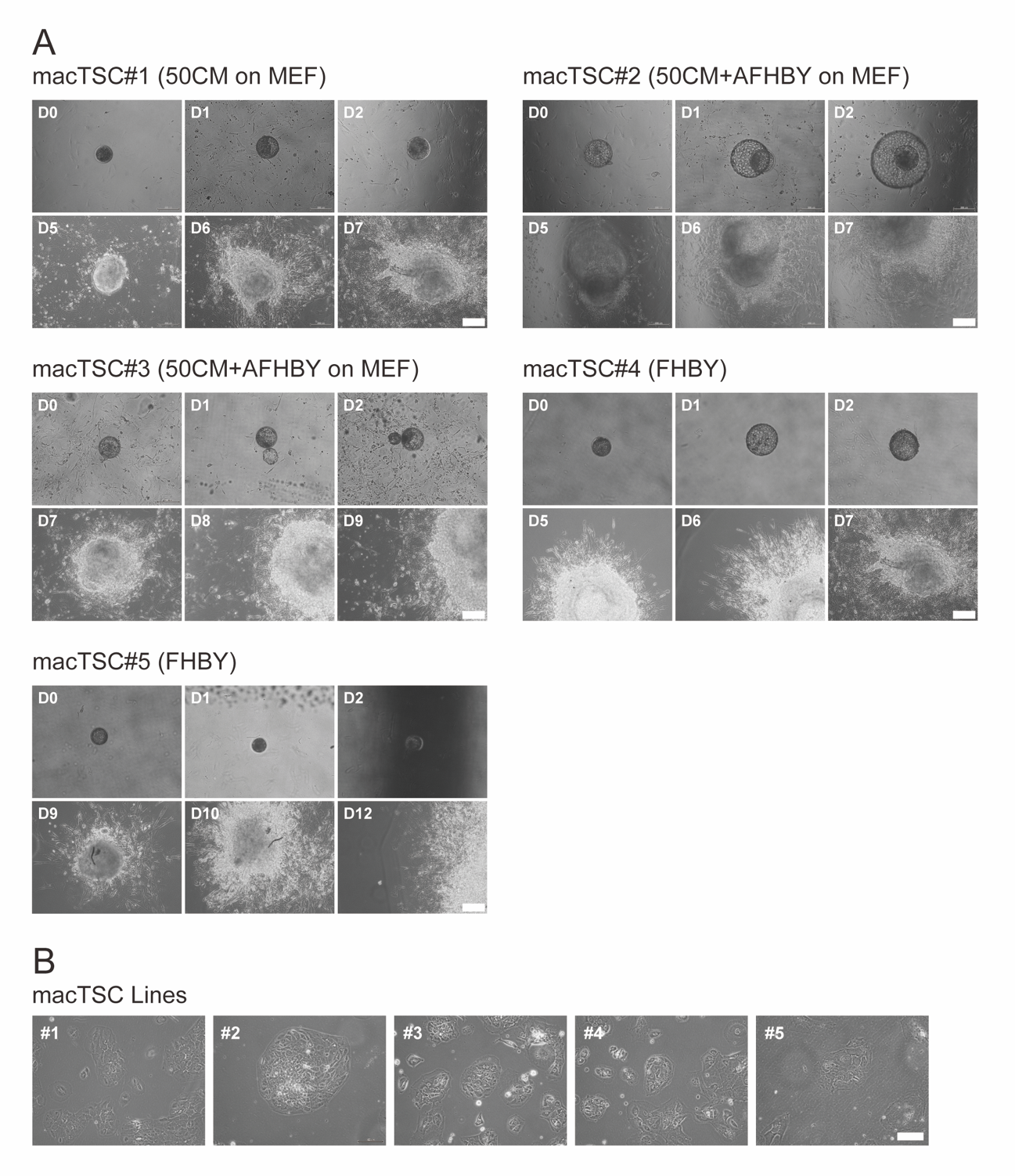


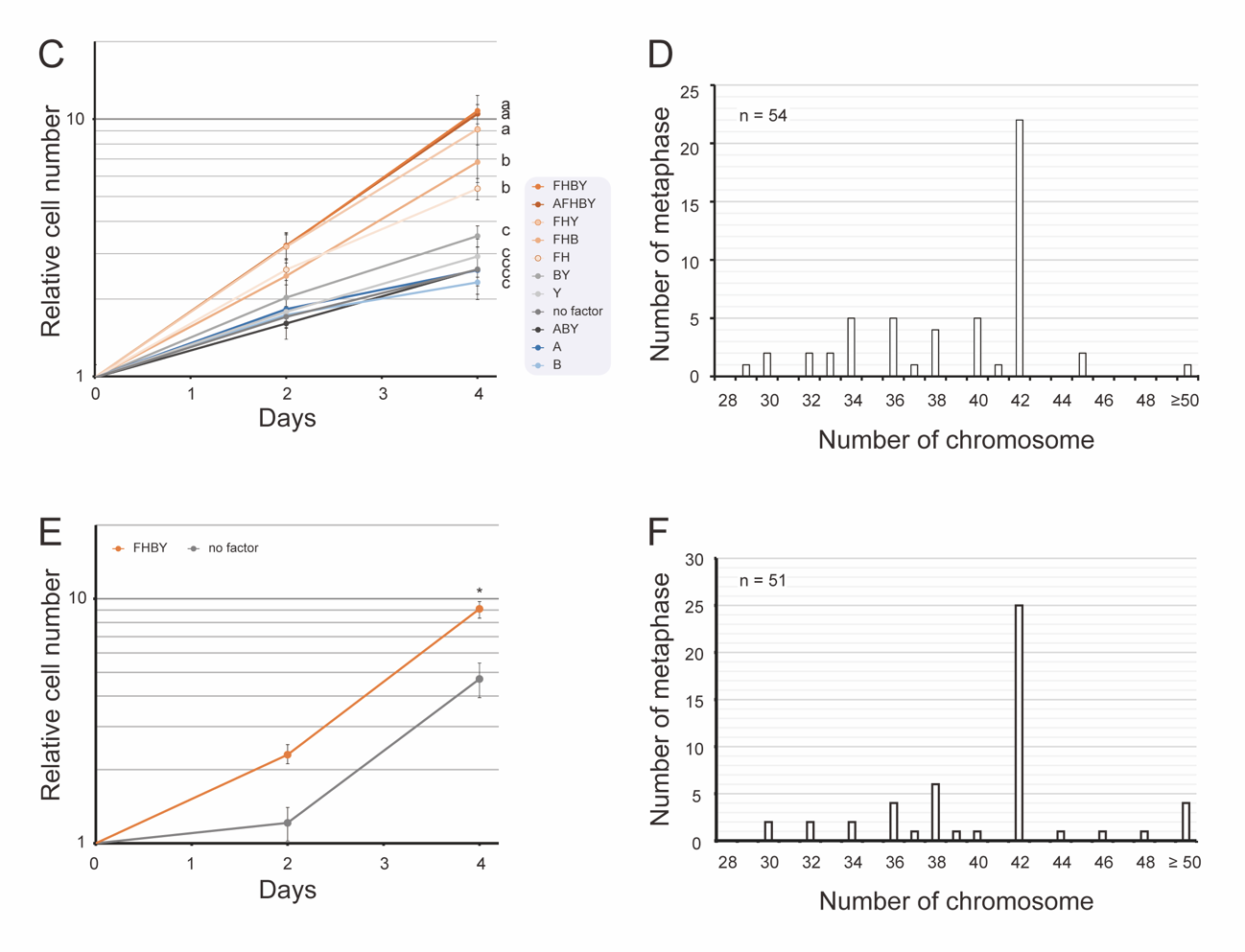


**Supplementary Figure S1.** Establishment of cynomolgus monkey trophoblast stem cell lines (macTSC) from blastocysts. (*A*) All of the blastocysts used in the present study displayed attachment by day 5 of cell culture (D5), formed cell outgrowth, and eventually gave rise to stably growing cell lines. See the main text for the specific culture conditions. Scale bar = 200 μm. (*B*) The appearance of the five macTSC lines. Scale bar = 200 μm. (*C*) Growth of macTSC#2 in different culture conditions; related to Figure 1C. (*D*) The number of chromosomes in macTSC#2. Chromosome spreads were prepared from the colcemid-treated cells, and the number of chromosomes in fluorescent images of 54 spreads were counted. (*E*) Growth of macTSC#1 line. (*F*) The number of chromosomes in macTSC#1. The fluorescent images of 51 spreads were counted.


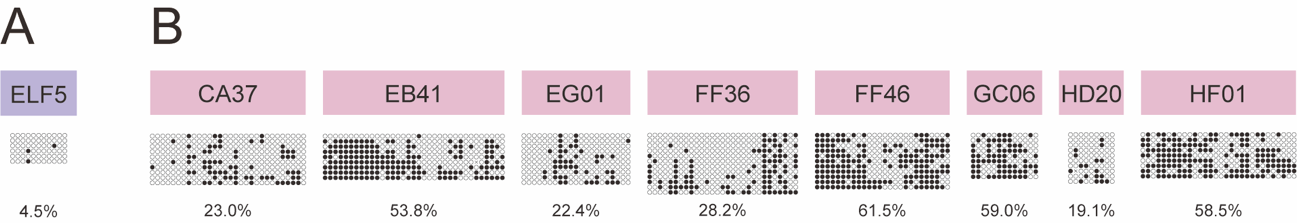


**Supplementary Figure S2.** (*A* and *B*) DNA methylation status of the *ELF5* promoter (*A*) in T-E T-DMRs (*B*) in macTSC#1. Open and filled circles represent unmethylated and methylated cytosines, respectively. Overall methylation percentage (the number of methylated CpGs per number of total CpGs) is shown under each part.


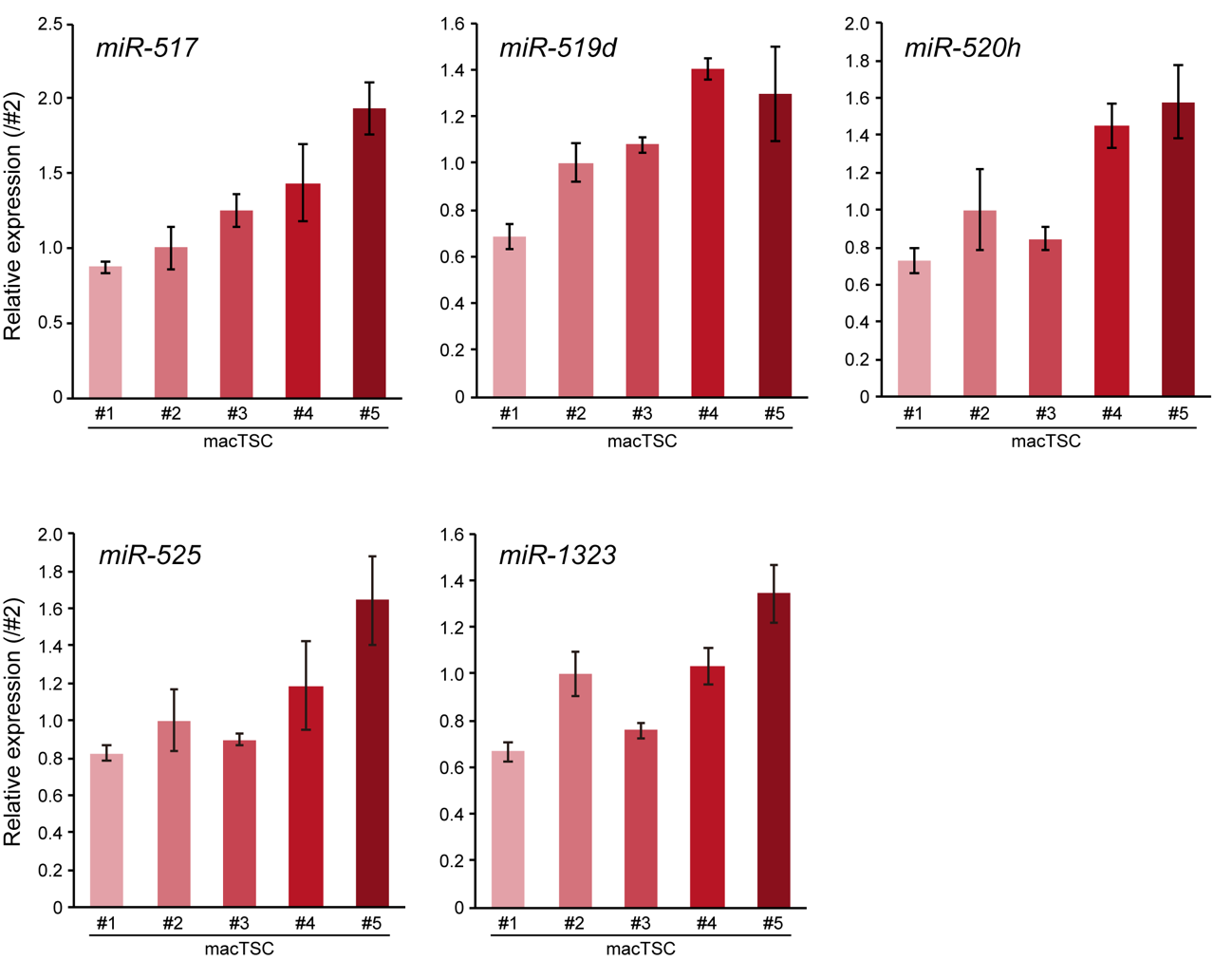


**Supplementary Figure 3.** The relative expression levels of five C19MC miRNAs in macTSC#1-5. The mean expression level (± SD) was normalized to that of U6 snRNA. Value of each miRNA in macTSC#2 was arbitrary set as 1. *; *p* < 0.05 (technical triplicates for biological duplicates).

**
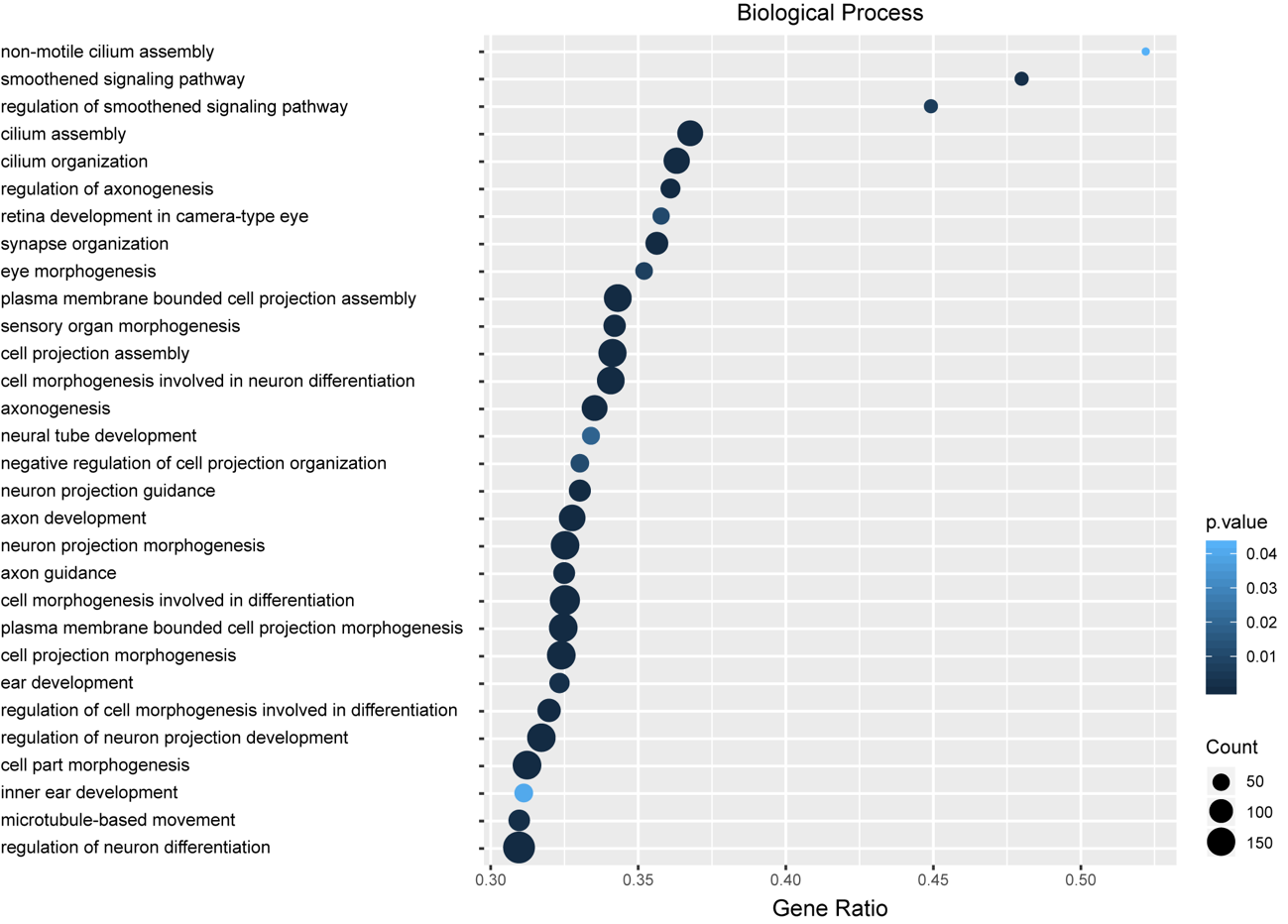
**

**Supplementary Figure S4.** The GO term enrichment analysis of the genes with < 2-fold lower expression in the all macTSCs than in ESC. For GO analyses, PANTHER (www.panther.org) was used. *P*-values were calculated by Fisher’s exact test and Bonferroni corrected.


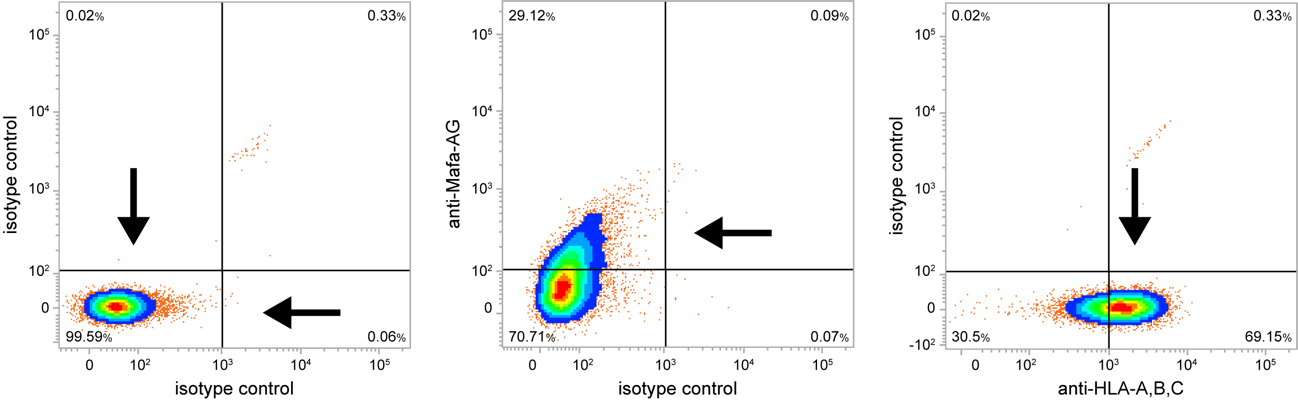


**Supplementary Figure S5.** Determination of the thresholds in flow cytometric analyses. The combination of antibodies and their isotype controls were used to draw threshold lines (arrows), so that a majority (> 99%) of double-negative controls distribute under the threshold lines.


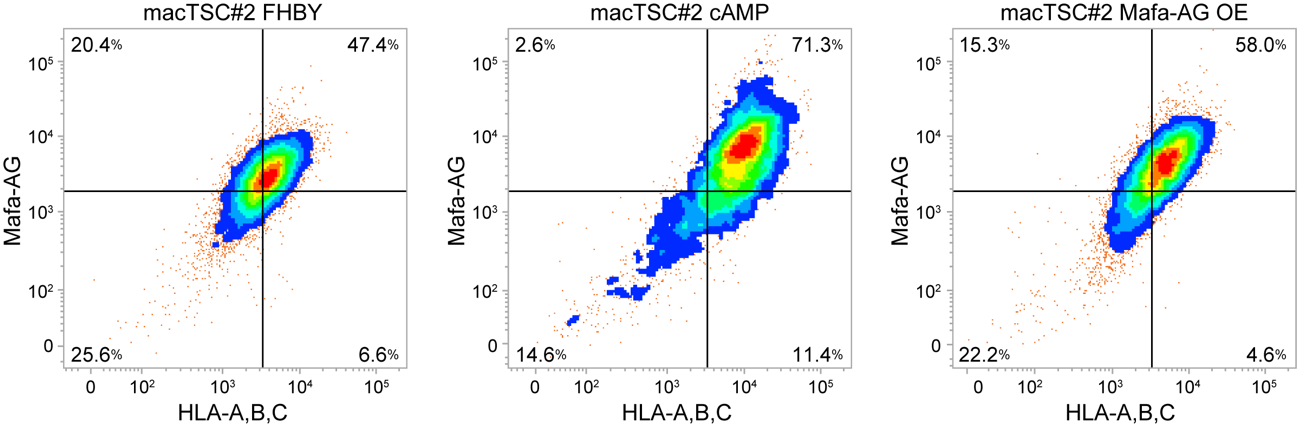


**Supplementary Figure S6.** Flow cytometric analysis in biological duplication; related to Figure 6C. PI staining-positive dead cells were gated out.


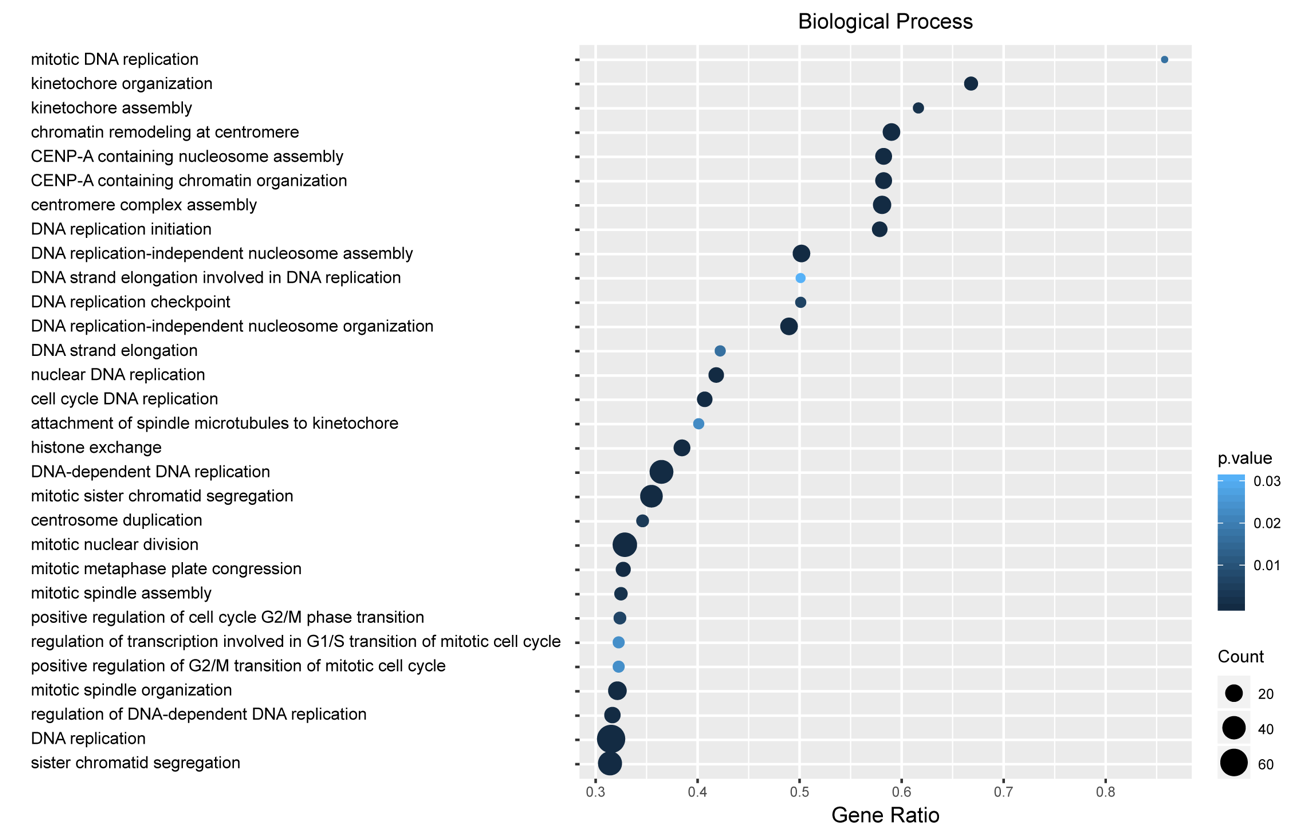


**Supplementary Figure S7.** The GO term enrichment analysis of the genes with < 2-fold lower expression in the all macTSCs in +cAMP than FHBY condition. For GO analyses, PANTHER (www.panther.org) was used. *P*-values were calculated by Fisher’s exact test and Bonferroni corrected.


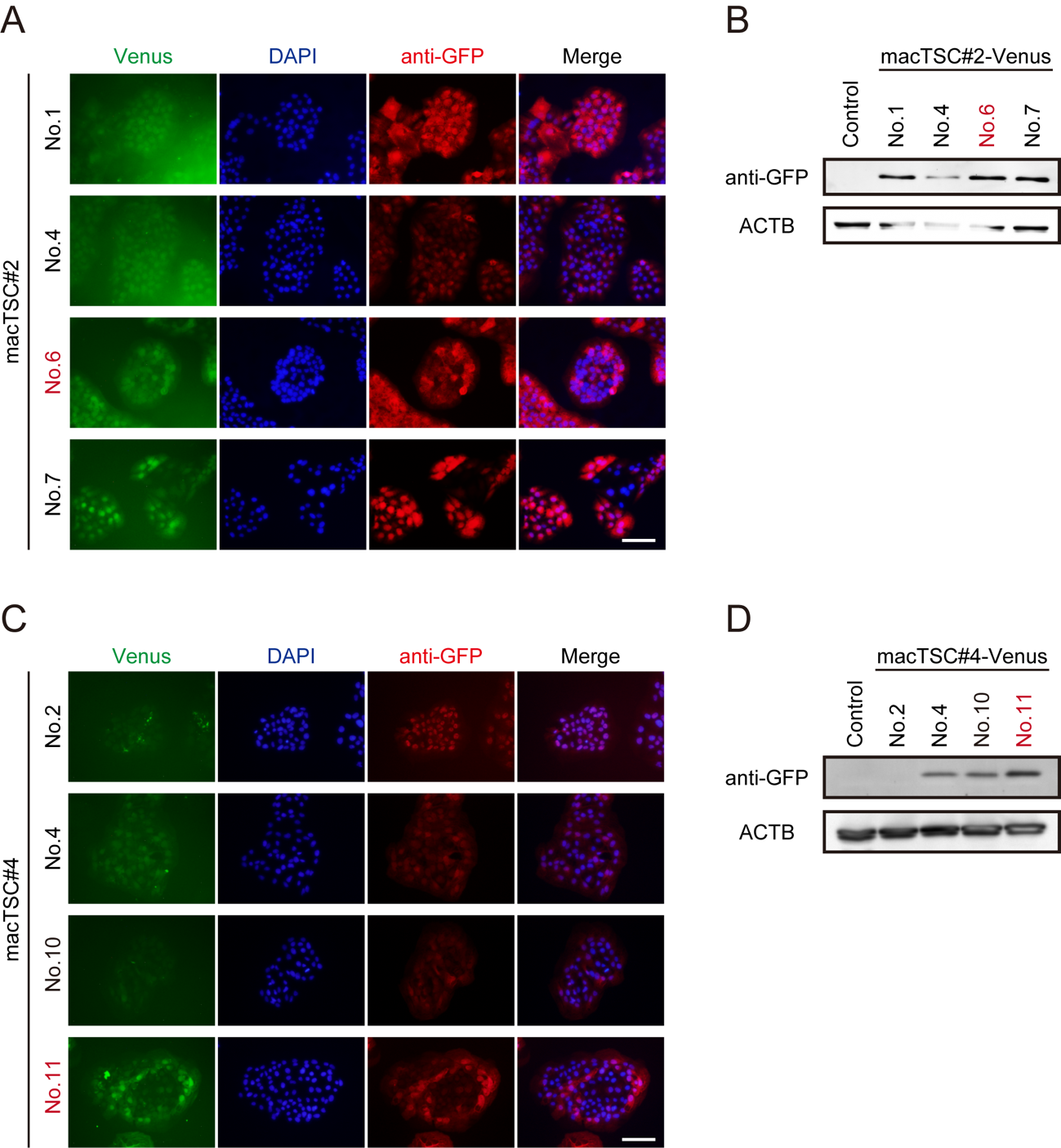


**Supplementary Figure S8.** Establishment of Venus-overexpressing macTSC lines. Detection of Venus in macTSC#2-Venus (*A* and *B*) and macTSC#4-Venus lines (*C* and *D*) by immunofluorescence (*A* and *C*) and western blotting (*B* and *D*). Scale bars in A and C = 100 μm. ACTB was used as an internal control for western blotting. Control, the original macTSC#2 (*B*) and macTSC#4 (*D*) cells. Based on the expression levels of Venus, macTSC#2-Venus No. 6 and macTSC#4-Venus No. 11 were chosen for the xenogeneic chimera assay.


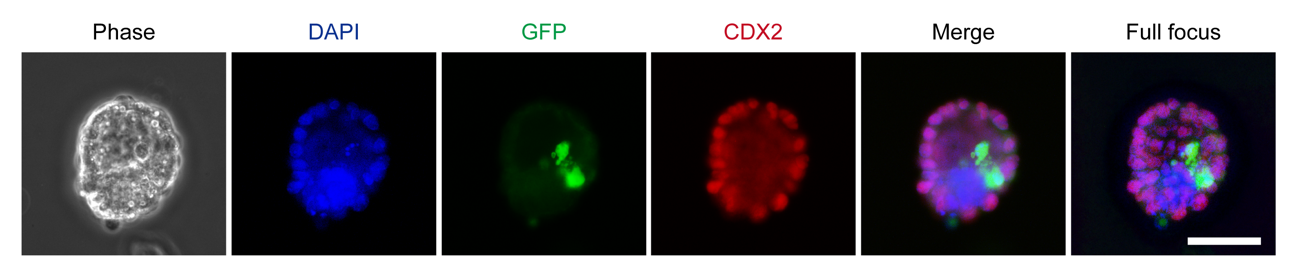


**Supplementary Figure S9.** Failure of aggregation in xenogeneic chimera blastocyst. macTSC#2-Venus cells dropped to the embryonic cavity. Scale bar = 50 µm.


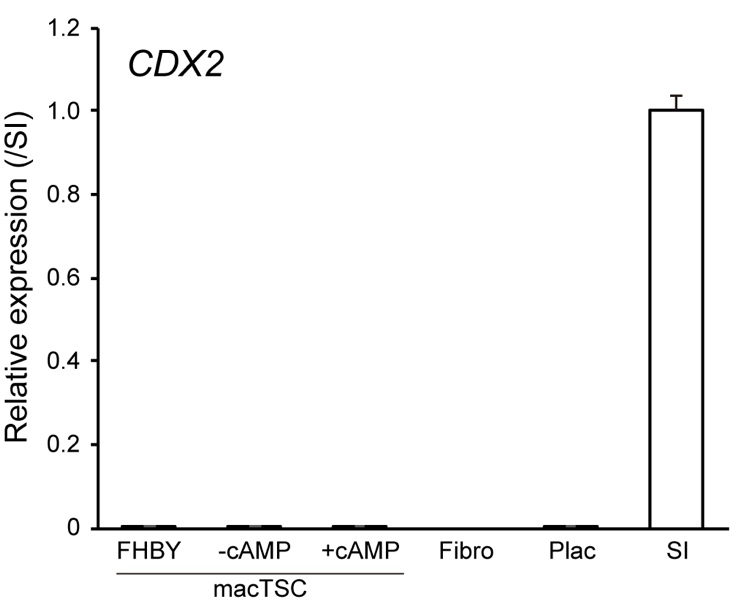


**Supplementary Figure S10**. qRT-PCR for analyzing the expression of *CDX2* in macTSC. The mean values (± SD) of technical triplicates for biological duplicates, normalized by the expression of *ACTB*, were shown relative to that of small intestine (SI; arbitrarily set as 1).


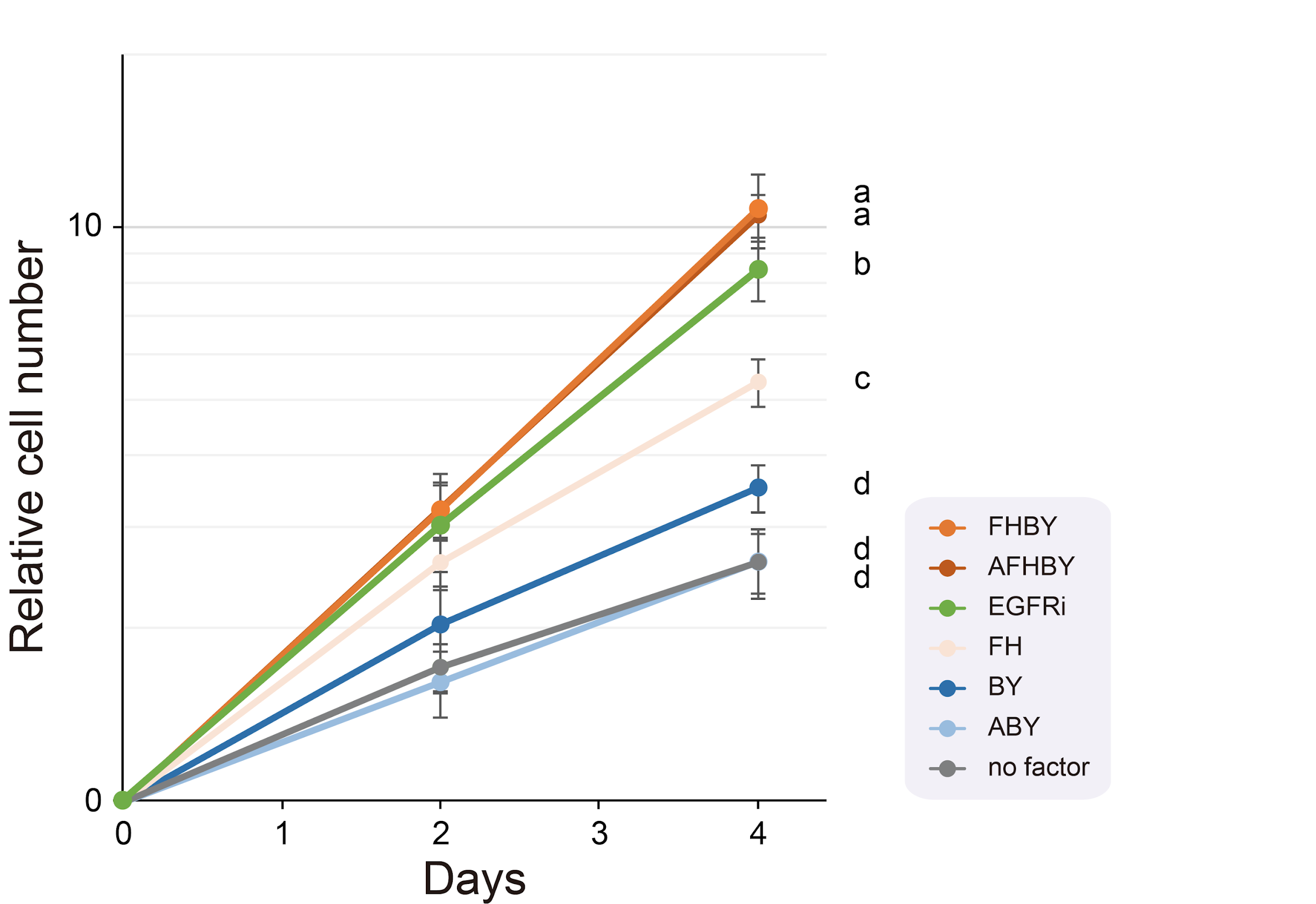


**Supplementary Figure S11.** Growth of macTSC#2 treated with 1 µM of EGFR inhibitor, PD153035 (EGFRi) (TCI, Tokyo, Japan); related to Figure 1D. The number of cells normalized to that at day 0 and arbitrarily set as 1. The mean values (± SD) of technical triplicates for biological duplicates are shown. *P* < 0.05 between different characters on day 4 (The Tukey-Kramer test).

**
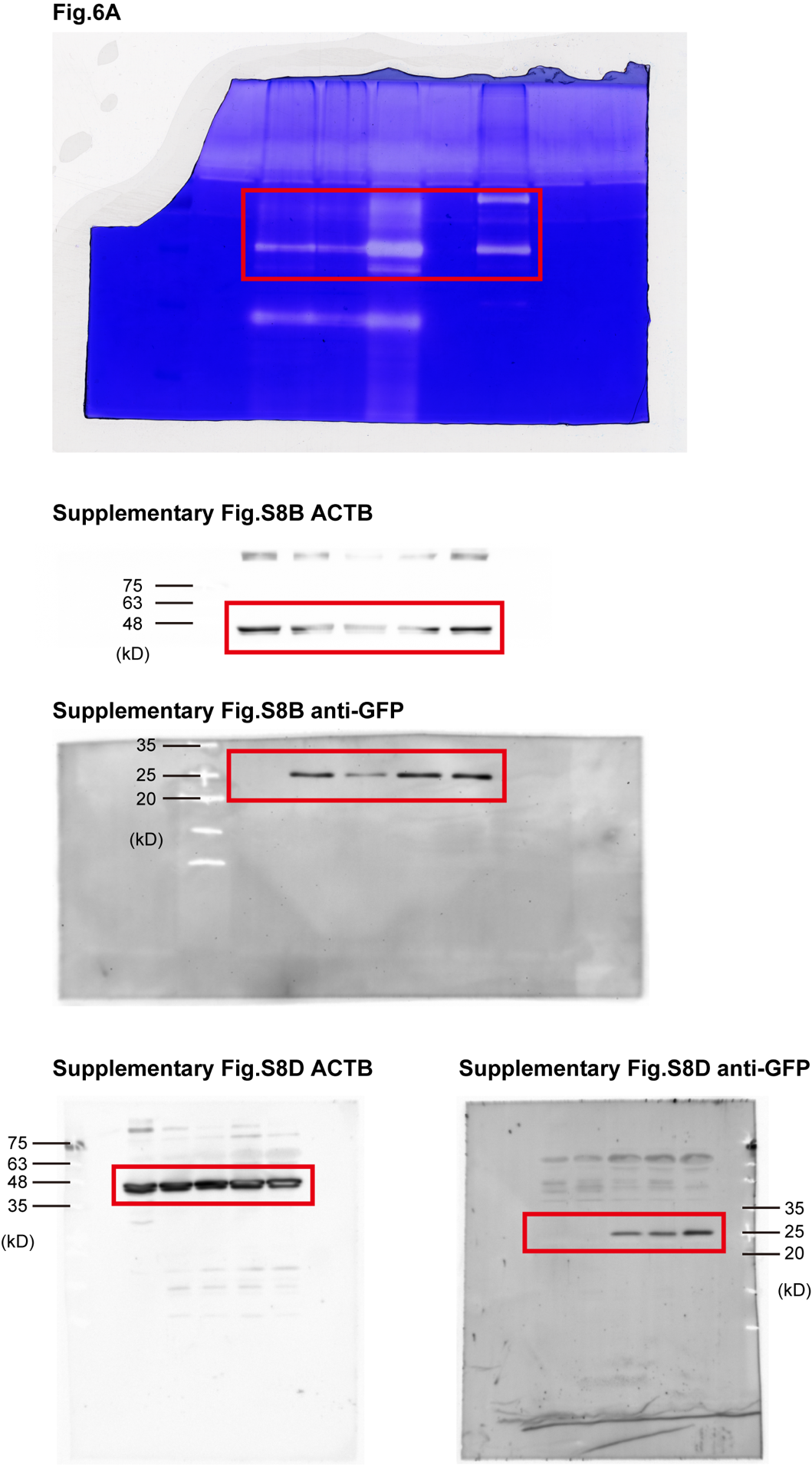
**

**Supplementary Figure S12.** Uncut blot images.
